# AffyMAPSDetector: a software tool to characterize Affymetrix GeneChip™ expression arrays with respect to SNPs

**DOI:** 10.1186/1471-2105-8-276

**Published:** 2007-07-30

**Authors:** Sunita Kumari, Lalit K Verma, Jennifer W Weller

**Affiliations:** 1 Department of Computer and Information Science, Indiana University Purdue University Indianapolis, Indianapolis, IN, 46202, USA; 2 Eli Lilly and Company, Indianapolis, IN, 46285, USA; 3 Computer Science/Bioinformatics program, University of North Carolina at Charlotte, Charlotte, NC, 28223, USA

## Abstract

**Background:**

Affymetrix gene expression arrays incorporate paired perfect match (PM) and mismatch (MM) probes to distinguish true signals from those arising from cross-hybridization events. A MM signal often shows greater intensity than a PM signal; we propose that one underlying cause is the presence of allelic variants arising from single nucleotide polymorphisms (SNPs). To annotate and characterize SNP contributions to anomalous probe binding behavior we have developed a software tool called AffyMAPSDetector.

**Results:**

AffyMAPSDetector can be used to describe any Affymetrix expression GeneChip™ with respect to SNPs. When AffyMAPSDetector was run on GeneChip™ HG-U95Av2 against dbSNP-build-123, we found 7286 probes (belonging to 2,582 probesets) containing SNPs, out of which 325 probes contained at least one SNP at position 13. Against dbSNP-build-126, 8758 probes (belonging to 3,002 probesets) contained SNPs, of which 409 probes contained at least one SNP at position 13. Therefore, depending on the expressed allele, the MM probe can sometimes be the transcript complement. This information was used to characterize probe measurements reported in a published, well-replicated lung adenocarcinoma study. The total intensity distributions showed that the SNP-containing probes had a larger negative mean intensity difference (PM-MM) and greater range of the difference than did probes without SNPs. In the sample replicates, SNP-containing probes with reproducible intensity ratios were identified, allowing selection of SNP probesets that yielded unique sample signatures. At the gene expression level, use of the (MM-PM) value for SNP-containing probes resulted in different Presence/Absence calls for some genes. Such a change in status of the genes has the clear potential for influencing downstream clustering and classification results.

**Conclusion:**

Output from this tool characterizes SNP-containing probes on GeneChip™ microarrays, thus improving our understanding of factors contributing to expression measurements. The pattern of SNP binding examined so far indicates distinct behavior of the SNP-containing probes and has the potential to help us identify new SNPs. Knowing which probes contain SNPs provides flexibility in determining whether to include or exclude them from gene-expression intensity calculations; selected sets of SNP-containing probes produce sample-unique signatures.

AffyMAPSDetector information is available at

## Background

Affymetrix manufactures high-density oligonucleotide microarrays for transcript measurement, a platform known as the GeneChip™. Each gene is represented on a GeneChip™ by one or more sets of oligonucleotide probe pairs that have been designed to capture transcripts from a biological sample. A probeset consists of 11–16 probe pairs; each probe pair is made up of a perfect match (PM) and mismatch (MM) 25-mer oligonucleotide. The sequence of the PM probe is designed to be an exact complement of a segment of a transcribed portion of the gene to which the probe maps and is intended to allow quantification of the transcription level of that gene. The corresponding MM probe differs from the PM probe by a single base substitution (the homomeric transversion) at the 13^th ^position. In the array design (chip information file) supplied by Affymetrix, the 13^th ^position is called either the mismatch or interrogation position of the probe sequence. The original intent behind incorporating a MM probe for each PM probe was to provide a sensitive control for the identification and quantification of non-specific hybridization as a source of background signals [1,2]. However, several factors, such as the physical accessibility of probe-target interaction sites under experimental conditions and cross-hybridization from other alleles and other genes, may contribute to the MM readout being higher than the corresponding PM value. The relative importance of these contributing factors is currently unknown. One of our research goals is to better understand and characterize the factors that may lead to anomalous probe values. In this study, we focus on characterizing the presence of Single Nucleotide Polymorphisms (SNPs) as a source of probe readouts that do not reflect transcript concentration levels, and we investigate the effects on downstream results when one does not compensate for such occurrences.

Hybridization of labeled target to any probe is due to both fully and partially complementary sequences. Therefore, measurable signal results from both hybridization of the probe to the intended target and cross-hybridization to an unintended target. There is a substantial body of publications reporting on the probable sources of cross-hybridization from alternate sites in the genome, but not from alternate alleles of the same locus [3] which is what SNPs represent. Removal of cross-hybridizing probes is based on sequence similarity comparison data, but is not data driven in the sense of using measurements of PM and MM levels to search for the responsible binding partners. In many experiments, the data shows that for a significant number of probes the MM intensity exceeds the PM intensity. Since this violates the assumptions of good probe behavior, the common analysis strategy in this case is to eliminate these probe pairs from the dataset [4,5]. While this is reasonable when the cause can be rigorously assigned to cross-hybridization, the reverse reasoning cannot be applied, that is, MM ≥ PM does not always result from cross-hybridization of sequences from different genes, and, as shown below, elimination of measurements based on this observation may incorrectly change the quantitation or call status outcome for particular genes.

In fact, the correct interpretation of MM data as a whole remains a topic of debate, and as yet there is no single consensus on how to handle background subtraction for Affymetrix microarrays. For instance, Zhou and Rocke [6] present a number of strategies addressing whether and when to use MM measurements as part of the background adjustment, but those strategies which do use the intensity of MM probes assume that non-specific hybridization is monitored. In the analyses described here, we chose to use certain procedures recommended by Affymetrix, the manufacturer of the data production platform. In brief, the Affymetrix probeset detection calling algorithm uses the MM intensity to estimate the stray signal. If the MM intensity is higher than the PM intensity, Affymetrix Microarray Suite 5.0 (MAS 5.0) flags the outcome as uninformative and computes an idealized version of the MM signal, which is then subtracted from the corresponding PM probe signal. This idealized version of a MM signal (IM) is always smaller than the corresponding PM signal [7]. To obtain the expression signal that is representative of a complete probeset, the MAS 5.0 algorithm compares signal intensities from the constituent PM and MM probes to classify the overall probeset measurement in a 'detection call' as being either 'Present' (P), 'Absent' (A), or 'Marginal' (M) [8]. The impact of MM signal handling on different analysis strategies will depend on their individual assumptions; for example, Robust Multi-chip Average (RMA) ignores any MM contribution to the expression intensity signal [9] and therefore will lose information when the alleles present bind most strongly to the MM probes.

Since a mismatch probe is the result of a single base difference between two sequences, the concept that probes might be detecting SNP alleles seemed a natural property to consider [10]. An individual might be heterozygous such that one allele binds to the PM and the other to the MM probe with high specificity and similar intensity (PM = MM), or homozygous for the allele matching a MM probe (MM > PM). Therefore, if not accounted for, the presence of two different alleles or the presence of only the allele complementary to the PM, will cause the relationship between the PM and MM pair to be inverted in the analysis, with subsequent misinterpretation [11]. In some cases neither the PM nor MM probe is the perfect complement of the allele present, in which case there will still be preferential binding that can lead to low levels of binding to the PM probe and/or high levels of binding to the MM probe [12]. The deviations from ideal behavior of the matched probe pairs can be predicted in these cases. As mentioned above, under these circumstances, the MAS 5.0 algorithm arbitrarily adjusts the MM value to ensure that it is less than the PM value, but given a flag indicating the presence of a SNP, MAS 5.0 could be modified to handle the PM and MM values appropriately; for example by swapping the two values if a SNP is present that makes the allele complementary to the MM probe, or removing the values entirely if correct genotype information is not available.

We identified the SNP-containing probes on the chip used for an experiment that had a large number of human samples (examined for expression but not genotyped for SNPs) and then examined differences between the bulk properties of intensity values and ranges for SNP-containing and non-SNP-containing probes. To test the effect of taking into account the possibility of a SNP in a probe on a specific gene in a particular sample, we also tested whether either the Presence/Absence call or the total intensity of a gene was affected by how we handled the probe. Because the number of individual probes on a chip is very large (in the hundreds of thousands), and the SNP-identification databases are updated fairly frequently, the process of mapping a SNP to a probe was automated to allow rapid, consistent and reliable whole-array screening.

A Java-based computational tool, AffyMAPSDetector, was developed to identify SNP containing probes in Affymetrix expression arrays. This allowed us to specifically screen for the intensity measurements due to probes that may contain SNPs, and investigate their properties with the goal of improving our data pre-processing methods and subsequent meta-analyses of the transcript-level data. While we have used SNP identification for processing existing expression datasets, this information can also be used in the design of probes and microarray experiments.

## Implementation

**Figure 1:**
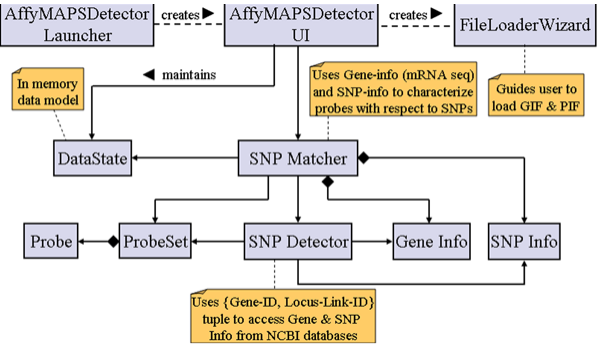
**AffyMAPSDetector architecture**. AffyMAPSDetector class diagram showing core domain concepts and relationships among them.

AffyMAPSDetector has been developed as a cross-platform desktop application using the Java technology suite from Sun Microsystems [13]. Its user interface is written in Java Swing and it requires JRE 1.4 or a higher version. The required JRE can be downloaded from the web-site at [13]. Before running AffyMAPSDetector as a desktop application, JRE 1.4 or higher must be installed and the computer must be connected to the internet. Figure 1 shows the core concepts and underlying architecture of the AffyMAPSDetector using the Unified Modeling Language (UML).

**Figure 2:**
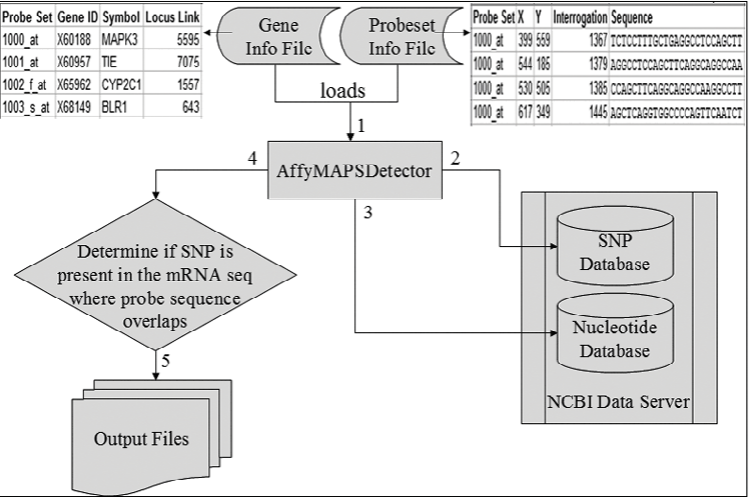
**AffyMAPSDetector process flow diagram**. AffyMAPSDetector process workflow to retrieve SNPs and map them to the corresponding probes. GIF and PIF are "stylized" for brevity, showing only critical columns (actual files have additional columns that are not shown here).

AffyMAPSDetector requires two ASCII text files as input data sources: "NetAffx Annotation File" and "Sequence File". Both of these files are available for download from the Affymetrix support page under "NetAffx Annotation File" and "Sequence Files" respectively [14]. Please note that Affymetrix requires registration before you can download the annotation files. Here we refer to the "NetAffx Annotation File" as the gene information file (GIF) and the "Sequence File" as the probeset information file (PIF). For the HG_U95Av2 chip, the current GIF and PIF files are available from the Affymetrix HG-U95 main support page cited above. The GIF appears in the "NetAffx Annotation Files" section as HG_U95Av2 Annotations, CSV (5.0 MB, 12/20/05) and the PIF appears in the "Sequence Files" section as HG_U95Av2 Probe Sequences, Tabular (2.9 MB, 1/27/06). The GIF file contains information at the level of the genes (including probeset name, gene-identifier, LocusLink ID (now Entrez Gene), gene-name, chromosome, gene description etc.) that are interrogated by the chip. The PIF file contains probe specific information including the probeset name, the probe's x and y coordinates on the chip, the interrogation (13^th^) position, the probe's sequence, and target strandedness etc. AffyMAPSDetector has a simple graphical interface that guides the investigator through the process of loading the GIF and PIF files and starts the process of SNP detection with the simple click of a button. Figure 2 shows the AffyMAPSDetector process workflow as it retrieves the location of SNPs and maps them to the probes in a given probeset. GIF and PIF files are stylized for brevity in Figure 2.

For each chip, AffyMAPSDetector builds a correspondence between the probeset and target sequence related information using the {"representative public id", "locus link"} tuple. For an i^th ^probeset, AffyMAPSDetector uses the corresponding tuple to extract the following information from NCBI SNP and nucleotide databases [15,16]:

• RefSeq mRNA sequence of the gene referenced by gene accession number.

• List of SNP locations, if present, in the mRNA sequence for the {"representative public id", "locus link"} tuple.

• For each SNP, SNP details including: alleles, heterozygosity, SNP class, SNP position at (+)/(-) strand etc.

AffyMAPSDetector determines the sequence correspondence between the probe and target sequence segment, including comparison of the starting, middle, and end positions of each probe of the i^th ^probeset with the corresponding mRNA sequence. It compares the probe's interrogation-position (based on the PIF) with the middle position in the matched mRNA segment. If the middle and the interrogation positions are not the same, the middle position is used as the reference 13^th ^position in the probe sequence. AffyMAPSDetector then uses the SNP-list for the i^th ^probeset to check for the presence of SNPs at all possible positions in the probes in the i^th ^probeset. Table 1 shows a subset of the SNP output file generated by AffyMAPSDetector using dbSNP as the source. Here, "Range-Min" and "Range-Max" correspond to the starting and the ending base-indices of the probe sequence with respect to the transcript sequence. Row number 4, corresponding to probeset *1341_at*, shows an instance of the "interrogation" position (575) that does not agree with the current "middle position" (786) of the probe. Since Affymetrix probes are designed against "exemplar sequences" derived from transcript and EST sequences, it is always possible that, as transcript and EST databases evolve, a lack of correspondence between the 13^th ^probe-position and the complementary mRNA segment will occur, leading to the lack of congruence between the probe and its intended target that we occasionally pick up.

**Table 1:** Sample of SNP Detection output from AffyMAPSDetector.

**Probeset**	**X**	**Y**	**Gene ID**	**Locus Link**	**Range Min**	**Interrogation**	**Middle Position**	**Range Max**	**Probe Sequence**
1260_s_at	451	467	M16594	2939	500	512	512	524	AAGACTACCTTGTTGGCAACAAGCT
1261_i_at	348	549	M16595	2939	872	884	884	896	TACAACTCCTATTCACCCACTTAGT
**1341_at**	**499**	**313**	**X52056**	**6688**	**774**	**575**	**786**	**798**	**AGGACAAGGGCACCTTCCAGTTCTC**
1347_at	341	471	S78187	994	2955	2967	2967	2979	GTCACAGAAGCAGCTAAACCAAGGA

A subset of SNP detection output file generated by AffyMAPSDetector run on HG-U95Av2. Some of the columns have been omitted for brevity. Range Min is the where the probe starts on the transcript and Range Max is where it ends. Row 4 shows an instance where the interrogation position differs from the mismatch (middle) position.

AffyMAPSDetector generates five tab-delimited ASCII text files and a log file. The type of information contained in each file is described below:

• *Probes_With_SNPs.xls *file contains probes that are determined to contain documented SNPs at any position in the probe sequence.

• *Probes_Without_Snps.xls *file contains the list of genes and probesets for which no SNPs are found.

• *Genes_Without_Locus_Link.xls *file contains the list of those genes for which either LocusLink information is not provided in the gene-information file or for which AffyMAPSDetector cannot parse the LocusLink as a positive integer.

• *Genes_Info_From_Web.xls *file contains the mRNA sequences of genes that are collected by AffyMAPSDetector from the NCBI nucleotide database and used for determining the probe-positions within mRNA sequences.

• *Snps_Info_From_Web.xls *file contains additional information about probes with SNPs at the 13^th ^and/or any other position. This includes: "Nucleotide Accession Number of Gene", "SNP position with respect to mRNA sequence", "dbSNP Reference Cluster ID – rs#", "Protein Accession Number", "Function", "SNP Class", "Heterozygosity", and "Allele".

• *Log.txt *file contains the output log messages resulting from AffyMAPSDetector execution. The log information is primarily useful for post-processing follow up, for example, in cases that are described by text such as "a given probe sequence was not found in the extracted mRNA sequence"; "program failed to extract mRNA sequence"; "SNPs not found for a given gene"; "program failed to find LocusLink for a gene" etc. This helps the user interpret the cause of missing values.

## Results

### AffyMAPSDetector results for dbSNP-build-123

AffyMAPSDetector can be used to characterize any Affymetrix Expression GeneChip™ with respect to SNPs if the underlying GIF and PIF files are set up appropriately. For the results reported here, the application was run against the NetAffx-supplied GIF and PIF files for HG-U95Av2 GeneChip™, the NCBI's SNP database (dbSNP-build-123) and the nucleotide database (GenBank/RefSeq). The GeneChip™ HG-U95Av2 contained a total of 199,084 probes belonging to 12,625 probesets (11,302 unique genes). Processing this chip using AffyMAPSDetector generated the five tab-delimited ASCII text files and log file described above. Output from AffyMAPSDetector for this array design run against dbSNP-build-123 is described below:

• *Probes_With_SNPs.xls *file: 7,286 probes were determined to contain documented SNPs. These probes belonged to 2,582 probesets (2,479 unique genes). To inspect this list, see additional file 1: 'Complete SNP output file'. Of the 7,286 probes, 325 probes had at least one SNP at the 13^th ^position and 6,961 probes had one or more SNPs at positions other than the 13^th ^position. To inspect this list see additional file 2: 'Probes having SNPs at mismatch location'.

• *Probes_Without_Snps.xls *file: The probes from 8,474 probesets (7,662 genes) did not have any documented (i.e. currently known) SNPs in the dbSNP- build-123. To inspect this list see additional file 3: 'Probesets without SNPs'.

• *Genes_Without_Locus_Link.xls *file: The GIF contained 1515 probesets (1,376 genes) that lacked LocusLink information. However, of the 1,515 probesets, AffyMAPSDetector was able to extract LocusLink information from the NCBI SNP website for 528 probesets (476 genes). Of the remaining 987 probesets (900 genes) on the chip, 146 genes were sourced from *The Institute of Genome Research *(TIGR), but without the corresponding NCBI Gene ID and locus link information it was not possible to query the NCBI server for related metadata. For the other 754 genes, LocusLink information was not available at the NCBI website at the time AffyMAPSDetector was run on HG-U95Av2. To inspect this list see additional file 4: 'Expression genotype'.

• *Genes_Info_From_Web.xls *file: This file contains mRNA sequences of 11,147 genes. To inspect this list see additional file 5: 'HG-U95Av2 genes mRNA sequence'.

• *Snps_Info_From_Web.xls *file: This file contains useful meta-information at the gene level, including "Nucleotide Accession Number", "GenBank GI Number", "LocusLink ID" and "Gene Description" corresponding to all 11,147 genes. This information is very helpful for subsequent analyses, such as examining the position effects of labeling strategies, or determining the exon membership of probes. To inspect this list see additional file 6: 'Additional SNP information for having SNPs'.

• *Log.txt *file: Sequences of the 15,269 probes belonging to 2,304 probesets (or 2,249 unique genes) that do not map to their corresponding genes' mRNA sequence at collected in this file. To inspect the results please see additional file 7: 'AffyMAPSDetector execution log'.

Recently, AffyMAPSDetector was run against the latest dbSNP version, build-126. Results obtained from dbSNP-build-123 and dbSNP-build-126 are summarized in Table 2. Result files from test HG-U95Av2 runs using dbSNP-build-123 and dbSNP-build-126, and for additional gene chips including HG-U133, MG-430A2, and RAE-230, using dbSNP-build-123, are available from the project home page.

**Table 2:** Output results from AffyMAPSDetector run on HG-U95Av2.

	**dbSNP -build-123**	**dbSNP-build-126**
Number of probes that contain documented SNPs	7,286 probes from 2,582 probesets (or 2,479 unique genes)	8,758 probes from 3,002 probesets (or 2,858 unique genes)
Number of SNP containing probes involving 13th position	325 probes	409 probes
Number of SNP containing probes NOT involving 13thposition	6,961 probes	8,349 probes
Number of SNP containing probes involving 13th position only	251 probes	332 probes
Number of SNP containing probes involving 13th position and atleast one more position	74 probes	77 probes
Number of probesets (or unique genes) without documented SNPs	8,474 probesets (or 7,662 unique genes)	9,450 probesets (or 8,533 unique genes)
Number of probes NOT mapped into their respective reference mRNA sequence	15,269 probes belonging to 2,304 probesets (or 2,249 unique genes)	16,753 probes belonging to 2,168 probesets (or 2,017 unique genes)

Comparison of the output results from AffyMAPSDetector run on HG-U95Av2 against dbSNP-build-123 and dbSNP-build-126.

We note that output results will change as dbSNP evolves and grows, and also that updates in the build files must be treated with caution. For example, one such update of the NCBI SNP database (dbSNP-build-124) caused a significant change in the results we obtained; in particular there was a marked decrease in the number of probes containing SNPs. Further investigation, and follow up with the curators at NCBI, identified the cause as an internal dbSNP error in transferring the data in the new build, and a recommendation to revert to dbSNP-build-123. Therefore, we strongly suggest that the users carefully date output files and include versions of the source databases and files used as references.

### Experimental data set

In order to determine whether there is any significant effect due to the presence of SNP alleles on the analysis of an experimental dataset, the SNP identification results from AffyMAPSDetector were used to characterize the behavior of SNP-containing probes in a specific data set. Here, we used a HG-U95Av2 based lung adenocarcinoma dataset to examine the impact of discriminating SNP-containing probes [17,18] from non-SNP containing probes. This dataset contained 190 CEL files corresponding to 139 unique lung adenocarcinoma positive samples (47 out of 139 had two additional replicates). DNA-Chip Analyzer (dChip version 1.3) [19,20] was used to retrieve PM and MM intensity values corresponding to all probes in the 190 CEL files and these values were stored in a single large file, called the "Lung Adenocarcinoma Probe Data (LAPD)", available on the authors' project Web page.

### SNPs and probe intensity distribution

Since the samples were not genotyped, we did not know what SNPs were present in a particular sample. At the same time, the SNPs in dbSNP must be present in a minimal frequency in the test population, so it is reasonable to assume that some of them occurred in this relatively large experimental sample. In order to determine whether SNP-containing probes as a group behaved similarly to the single-allele detecting probes, we compared the intensity distribution of expression values for the following two sets of 325 probes:

1. Probes with SNPs: This group included 325 probes that had a SNP at the 13^th ^position.

2. Probes without SNPs: Probes in this group were randomly selected from the set of probes without any known SNPs. Several such sets were created, with similar outcomes each time.

**Figure 3:**
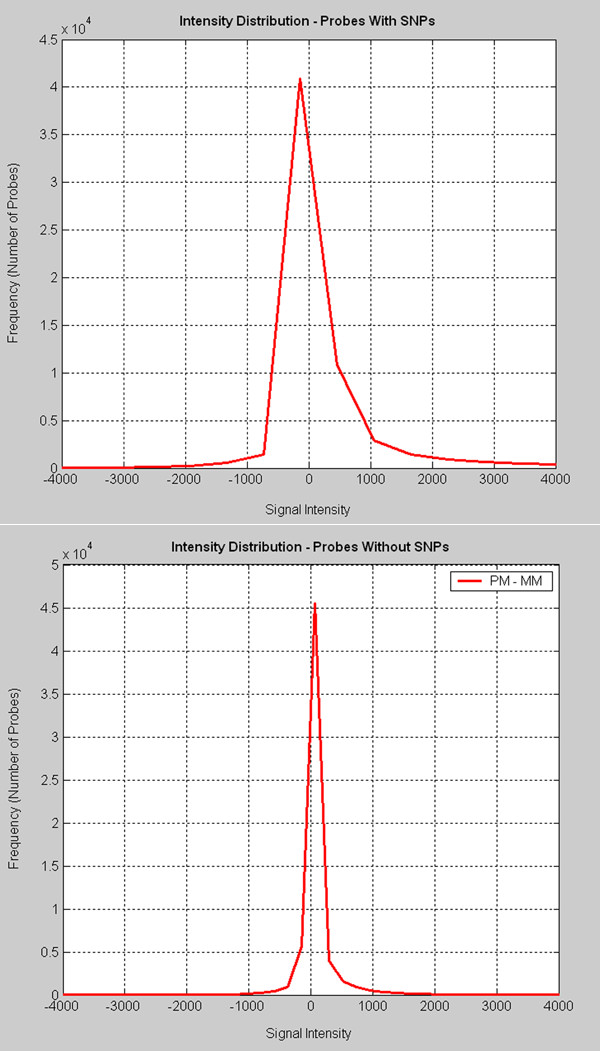
**Intensity distribution comparison of probes with and without SNPs**. A) Intensity distributions of HG-U95Av2 probes with SNPs (a set of 325 probes). B) Intensity distributions of HG-U95Av2 probes without SNPs (a set of 325 probes).

For each group, the intensity differences i.e. the (PM-MM) values were extracted from LAPD into a two dimensional matrix of size 325x190. The values were plotted using Matlab v7 (Mathworks™) and the distributions compared for mean and standard deviation from the mean. As seen in Figures 3A and 3B, intensity frequency distributions of these two datasets indicate distinct differences in both the mean and variance of the distributions of these two groups.

### SNPs and PM and MM probe binding efficiency

**Figure 4:**
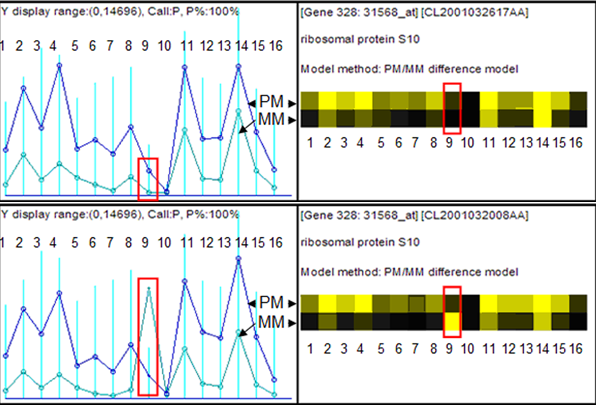
**Effect of SNP on PM and MM probe binding efficiency**. The left panels show the perfect match (PM, in blue) and mismatch (MM, in green) whereas the right panels show another view of PM (top row) and MM (bottom row) values across the probes in the same probe-set 31568_at and for the same gene S10. Probe 9 (from left to right), SNP containing probe, is the probe of interest, highlighted in red bounding box. A) For the sample – CL2001032617AA, dChip output for the gene S10 shows better binding of probe 9 with PM [P_9_(PM) ] as compared to MM [P_9_(MM)]. B) For the sample – CL2001032008AA, dChip output for the gene S10 shows better binding of probe 9 with MM [P_9_(MM)] as compared to PM [P_9_(PM)].

To explore the effect of SNPs on target binding efficiencies for PM and MM probes, we decided to compare adjacent probes on a transcript, where one probe was predicted to contain a SNP and the second was not. We initially focused on probes with one SNP at the 13^th ^position since they provided the least ambiguous case for analysis. However, in order to assess global properties we later expanded the criterion to include all SNP-containing probes. For a measurement to be valid the intensity must fall within a particular scanner range. Therefore, we selected the probes for which the PM and MM signal intensities in the 190 CEL files satisfied the requirement that the intensity fell in the linear measurement range, i.e. in the range [200 to 30,000 fluorescent units]. Results show a number of genes for which there was a great similarity in PM and MM values across all probes in a probeset, except at a SNP-containing probe. One such example is shown in Figures 4A and 4B, using dChip graphical output to compare and contrast the possible effect of a SNP on the expression levels of human ribosomal protein *S10*. The results for two different samples are shown: Sample-1 (AD249T1_A165_4; CL2001032617AA) and Sample-2 (AD335T2_A281_10; CL2001032008AA) from the lung adenocarcinoma study. In this figure it can be seen that for 15 of the 16 probes in probeset *31568_at *(which represents *S10 *on this chip) the intensity of PM>MM, and the intensity difference (PM-MM) in each probe is consistent between the two samples. This consistency is violated for the ninth probe, where SNP results from AffyMAPSDetector indicate that the ninth probe (P_9_) in probeset *31568_at *has a SNP at the 13^th ^position. One consequence is that, since dChip output for the gene for Sample-1 shows probe 9 with PM>MM intensity, it includes the P_9 _response in the computation of the 'Presence' call as well as in further downstream gene expression data analysis (Figure 4A). However, dChip output for this gene for Sample-2 has MM>PM intensity for probe 9, so it will not be used in the computation of the Presence call, and the intensity contribution will be replaced by one using an idealized background calculation, which modifies the total concentration calculation for the second sample (Figure 4B).

For more examples of genes in which probepairs showed this behavior in samples in this experiment, please see additional files 8 and 9: 'Behavior of SNP-containing probes with respect to PM and MM binding efficiencies.'

### Alteration of probeset detection call using PM/MM swap

Despite the example above, it is possible that a small change in intensity in one probe pair, averaged over 16 probe pairs, would have a very minor impact on the outcome, reflected either in the total intensity of the gene or in the Presence/Absence call of the gene. For this analysis we focused on the subset of SNP-containing probes that have a SNP at the 13^th ^position. For a randomly chosen sample (CL2001031611AA), the corresponding CEL file was modified by swapping PM and MM values for those samples where the probes containing SNPs had MM > PM, to study the effect on Presence/Absence calls of the genes. The program dChip was used to explore the effect of this exchange on the probeset detection call. To make the comparison, both the modified CEL file and the original (unmodified) CEL file were loaded into dChip simultaneously, thus simulating a comparison of two different samples/experiments. We found that, for some probesets, allowing for the presence of SNPs at the 13^th ^position by using the MM intensity in place of the PM intensity altered the probeset detection call (changing it from 'Absent' to 'Present' or 'Marginal' to 'Present'). In particular, the detection call for probesets *1486_at *and *34345_at *changed from 'Absent' to 'Present' and for the probeset *37746_r_at *the result was a change from 'Marginal' to 'Present'. To see the complete list of results see additional file 10: 'Examples of probes affecting probeset detection calls.' In these cases the genes will now have to be included in gene lists for downstream analyses such as differential expression estimation and clustering.

### Expression signature pattern analysis

**Figure 5:**
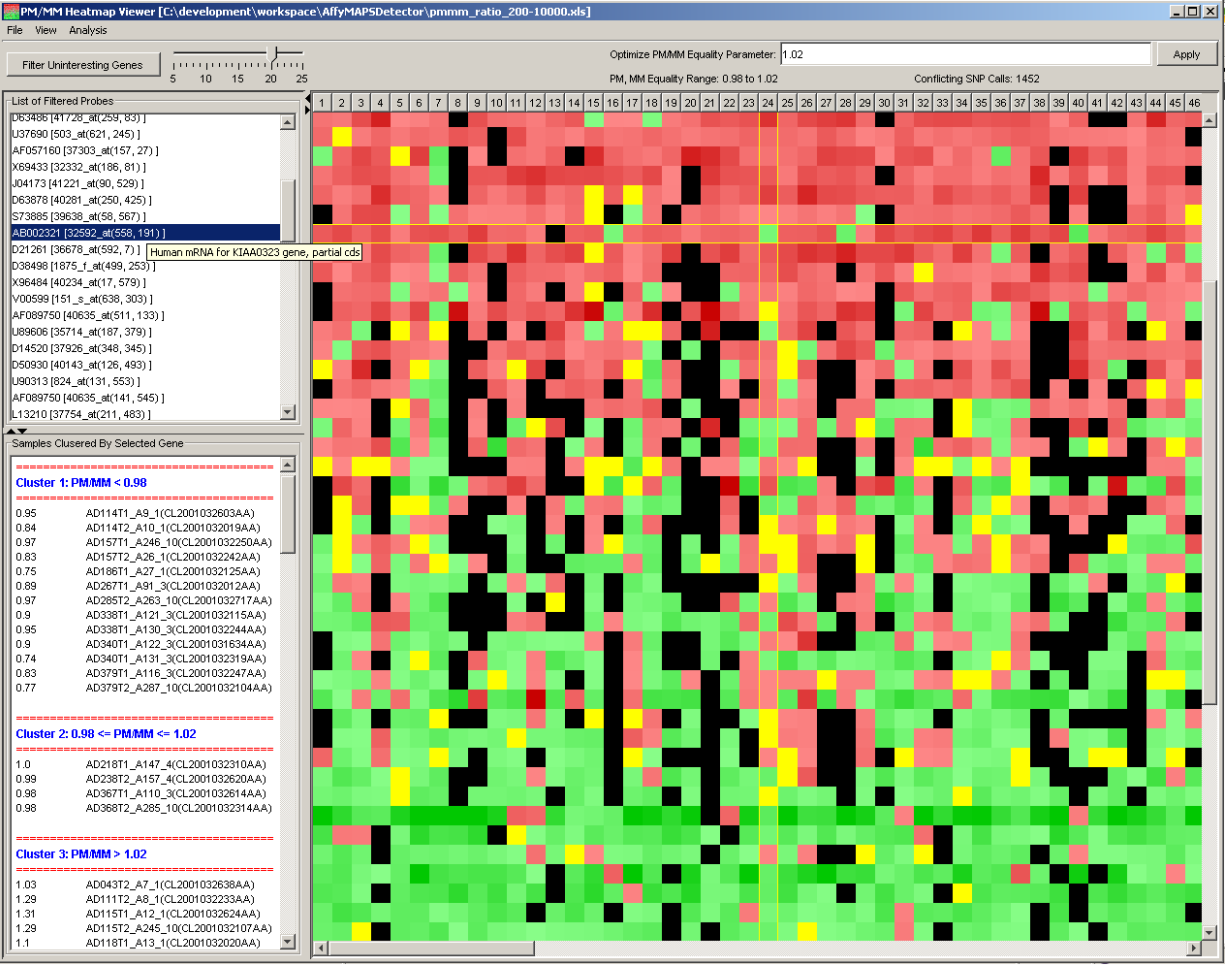
**Expression Genotype**. An instance of a PM/MM ratio data file from adenocarcinoma positive sample-replicates loaded into the heat-map viewer for expression signature pattern analysis. Removal of unchanging probes from the expression signature pattern and tuning of an equivalency parameter (E) to adjust for measurement precision differences leaves a dataset that can be classed as A [PM/MM < (1.0 - E)], class B [PM/MM > (1.0 + E)] or class A/B [PM/MM between (1.0 ± E) inclusive]. On the right, the table shows the data state as it goes through expression signature pattern steps.

The most common use of SNPs is to genotype individuals. The types of measurements available from gene expression arrays make it difficult to extract genotype information because expression level differences of each allele are layered on the genotype differences. It was not clear whether or not a sample genotype expression signature would emerge from GeneChip data. Since the ratio of PM to MM values was to be used and dividing by a small number inflates such a ratio, in the next experiment we required that intensity levels of both the PM and MM probes be at least 200 fluorescent units. We organized PM/MM ratio values as an [*n *× *m*] matrix in a tab delimited ASCII text file, where each row corresponded to a probe/gene of interest (i.e. a SNP-containing probe) and each column corresponded to a sample/chip of interest. We tuned for measurement reproducibility based on duplicate samples present in the experiment, and then binned the results into three categories, PM/MM ≥ 1, PM/MM ≈ 1, PM/MM ≤ 1. A heat-map type viewer was used for visualization of the results as shown in Figure 5; ratios of PM/MM > 1 are represented in red, PM/MM < 1 are represented in green, and PM/MM ≈ 1 are represented in yellow, with increasing color saturation as the ratio values increase. After removing the probes/genes that do not vary across the sample population, three classes of interest were identified:

1. Class A: samples in which "allele A is over-expressed compared to allele B."

2. Class B: samples in which "allele B is over-expressed compared to allele A."

3. Class A/B: samples in which "alleles A and B are equally expressed."

Based on the patterns of the classes, it was possible to select a subset of SNP-containing probes that uniquely identified each sample; reproducibility was confirmed by using duplicate samples as the test set. All duplicates were correctly identified and none of the unique samples had exactly the same pattern. See additional data file 11: 'SNP-containing probes' PM/MM ratio data file for expression genotype' as an example of the dataset that was used for expression genotyping.

### Software testing

AffyMAPSDetector was run on a Windows XP (Professional version 2002) platform with 1.6 GHz Intel^® ^Pentium^® ^M processor and 2GB RAM, on a Windows 2000 platform with 1.8 GHz Intel Pentium processor and 1.5GB of RAM, and on a second Windows 2000 platform with 1.6 GHz Pentium processor and 1 GB of RAM. It was observed that in each case AffyMAPSDetector took about six hours to finish processing 12,625 probesets housed on HG-U95Av2. Actual execution time will vary depending on factors such as the machine's processing power, size of the chip, internet bandwidth, and network traffic to the NCBI server. AffyMAPSDetector compiled and source codes are available as: additional data file 12 'AffyMAPSDetector v1 distribution package (compiled code)', and additional data file 13 'AffyMAPSDetector v1 source code'. These files are also available for download from the authors' project Web page.

## Discussion

The analysis of Affymetrix GeneChip™ expression arrays is quite complex because of the various factors contributing to the intensity measurements. Among the most commonly explored problems are those of cross-hybridization caused by other sites in the genome [21] and probe assignment inconsistencies that occur over time due to changing gene annotations [22]. Here, we have identified probes that are a potential source of signal assignment errors, either due to changes in the underlying sequence that we have identified, or due to the presence of alternate alleles arising from SNPs. Our software identifies:

1. Probes that no longer provide measurements for the gene that they were designed to represent.

2. Probes that measure targets known to have one or more SNPs, leading to the potential for confounding the PM and MM signal intensities and the relationship between the two in a sample.

AffyMAPSDetector results show that 15,269 (~7.7%) of the HG-U95Av2 probes do not actually map to the corresponding gene sequence. Table 3 shows the summary statistics of such probes for human, mouse, and rat gene chips. While not the AffyMAPSDetector's primary focus, the supporting data is collected during the normal course of processing, and can be used to compile the corresponding statistics by parsing the output log. These probes may be simply flagged and excluded from analyses or may be a source of interest in themselves. Here we have chosen to examine the effect of excluding them, since the focus of our interest was to study the effect of SNPs on the interpretation of gene intensity values. Of the remaining ~92.3% probes, ~3.7% (7,286) contain dbSNP-characterized SNPs. Among these, one group of 325 probes has SNPs present at the 13^th ^position while the other group of 6,961 probes has one or more SNPs at some position other than the 13^th ^position. The first group provides a simpler set for interpretation of the experimental results, since in this case the SNP position coincides with the mismatch position between the PM and MM probes.

**Table 3:** Inconsistencies in probe to gene annotations.

**Gene Chip**	**Total no. of probes**	**Total no. of un-mapped probes**	**Un-mapped probes belonging to**		**% Un-mapped probes**
				
			**Total no. of Probe sets**	**Total no. of Genes**	
HG-U95Av2	199,084	15,269	2,304	2,249	7.67
HG-U133A	247,965	7,735	1,853	1,842	3.12
MG-430A2	249,958	7,237	1,538	1,351	2.9
Rat-230A	175,477	1,999	559	543	1.14

Probe level statistics based on AffyMAPSDetector log results for probes that do not actually represent the gene that they were designed to represent (un-mapped probes).

In Figures 4A and 4B, where dChip graphical output was used to compare and contrast the possible effect of a SNP on the expression levels of human ribosomal protein *S10 *in two different samples, dChip flags P9 probe as an outlier (assuming that anomalous binding is due to either signal-saturation or cross-hybridization) and replaces the actual MM signal intensity value with a computed value in determining the 'Presence' call. This may adversely affect the quality of subsequent downstream analysis since the intensity value assigned to the gene changes as well. Although other researchers have produced lists of cross-hybridizing probes using different criteria for an acceptable match [21] and this probe did not appear on those lists, we independently checked for cross hybridization with transcripts from genes other than *S10*. We examined the BLAST hit results for the P_9 _sequence against the NCBI non-redundant nucleotide database "nr". The BLAST results indicate that the P_9 _sequence has no significant cross-hybridization with currently known expressed sequences. Therefore, for Sample-2, the observation that P_9_(MM) > P_9_(PM) can be best explained by the presence of a SNP at the 13^th ^position in this sample. Given that multiple alleles are represented in the population of samples, there are several associated consequences. First, the gene-quantitation results for the first sample will be altered by the intensity value contributed by this probe (for example, alteration in the results of fold-changes and clustering in the intensity-based methods). Second, a comparison of the gene expression between these two samples will be altered. Third, a potentially useful piece of information about the presence of a genotypic variant will be lost.

If SNP-containing probes are generally different in their behavior from non SNP-containing probes, then genes with such probes might cluster differently than they otherwise would based solely on the intensity differences. We quantitatively analyzed the intensity distribution of probes using the (PM-MM) difference that is customary when MM values are taken into account. The intensity profiles differ in both mean and variance, indicating that different properties are being measured by the two types of probes and therefore significant information may be lost if the analyst ignores SNP-containing probes in this dataset. The intensity values in the probes with SNPs were spread over a much wider range and the overall distribution was shifted towards negative values when compared to the intensities of the set of probes without SNPs, implying that the MM signal is greater than the PM signal for the 'average' probe pair in this group. In this dataset, the impact of SNP-containing probes is to decrease gene intensity estimates for genes that have such probes as compared to the genes without such probes. Unless samples are SNP qualified, our recommendation for the downstream intensity computation algorithms such as MAS5 and RMA is to remove the SNP-containing probes. In our analysis, we confirmed the repeatability of observed intensity distribution profile difference between the probes with/without SNPs by generating plots similar to Figure 3 for three independent selections of non-SNP containing probes. These results are contained in additional file 14: 'Intensity distribution profiles confirmation.'

Although the presence of SNP-containing probes has the potential to degrade the quality of gene expression data, we considered whether these measurements might be useful in their own right. Simultaneous (but non-identical) expression of both alleles of a gene has been observed by others [12]; we were interested in determining whether it is possible to identify allelic expression signatures of the samples using SNP-containing probe pairs. We performed an analysis to build allelic expression signatures using only those probes containing SNPs and showed that duplicate samples could be uniquely identified based on simple binning of expression ratios. We are in the process of developing a second program to perform more complete expression genotyping analyses, including a component for compensating for the different types and frequencies of SNP alleles.

## Conclusion

We present a tool that can be used to supplement the annotations provided for probes on the Affymetrix GeneChip™ platform. The SNP detection results from AffyMAPSDetector can be effectively used in the data analysis phase of a microarray experiment. In our study, the SNP results were found to be significant since the SNP-containing probes show behavior consistently distinct from the non-SNP containing probes when evaluated in terms of both the intensity distribution of each probe class and the contribution each class of probe has on the determination of individual transcript presence or intensity by several programs. The SNP annotation information can be used by researchers to assign a physical reason for the measured behavior of some of the MM probes. This information permits researchers to choose explicitly whether and how to include intensity estimates from these probes in the overall gene expression value for a probeset. Since SNP-containing probes behave differently from those without SNPs, separating the two for a given analysis may improve gene association and disease classification studies. As investigators use microarray experiments to study the intricate relationships and complex interactions between the molecular species in a biological system, we believe that the corresponding data-analysis or data-mining strategies will require processing of the data using multiple approaches. We have demonstrated the importance of one such approach in the interpretation of results from our research and we hope that it will serve others in a similar fashion.

We are currently in the process of extending the AffyMAPSDetector tool to produce explicit information about the cross-hybridization potential for every probe across the target genome including any characterized sequence variants, incorporating Nearest Neighbor estimates for the stability of all SNP-based alleles. We are also extending the AffyMAPSDetector utility set to allow assessment of GeneChip™ probe layouts in order to locate the probes with SNPs, probes without SNPs, any error prone probes that are not found to map to the corresponding gene's sequence, and potentially cross-hybridizing probes on the chip along with integrated context-sensitive annotations.

AffyMAPSDetector code and documentation distribution is open source under the GPL license and is available on the project home page. It can be readily modified to run locally if appropriate databases are set up correctly. This approach will allow the user to include proprietary information about SNPs in the analysis. We recommend regenerating the AffyMAPSDetector output files when source databases are updated. We have posted copies of the files generated for this report at the project home page. These can be downloaded by anyone interested in using the information to flag probes present in Affymetrix GeneChip designs without running the program. Although all reasonable efforts have been made to ensure the accuracy and reliability of the software and data, the changing nature of data sources and user specific configuration make it impossible for the authors to warrant the performance and/or results that may be obtained by using the software or data. The authors disclaim all warranties as to performance, merchantability or fitness of output of the software for any particular purpose. In any work or product derived from this material, proper attribution of the authors as the source of the software or data should be made.

## Availability and Requirements

• **Project name: **AffyMAPSDetector

• **Home page: **

• **Operating system(s): **Platform independent

• **Programming language: **Java

• **Other requirements: **Sun Java™ 2 Runtime Environment (JRE), Standard Edition, 1.4+; 256 MB or Higher RAM; about 300MB of free hard disk space.

• **License: **GNU GPL 

• **Availability: **Source code available from the project home page.

• **Any restrictions to use by non-academics: **None

## Abbreviations

AffyMAPSDetector: **Affy**metrix **M**icro**a**rray **P**robe **S**NP **Detector**

ASCII: American Standard Code for Information Interchange

CDF: Chip Definition File

dbSNP: NCBI SNP database

GIF: NetAffx Gene Information File

GUI: Graphical User Interface

IM: Algorithmically computed Ideal Mismatch intensity value

LAPD: Lung Adenocarcinoma Probe Data (dataset from lung adenocarcinoma study)

MAS 5.0: Affymetrix Microarray Suite version 5

MM: Mismatch Probe

PIF: NetAffx Probe Information File

PM: Perfect Match Probe

RMA: Robust Multi-chip Average

SNP: Single Nucleotide Polymorphism

UML: Unified Modeling Language

## Authors' contributions

JW formulated the main idea of systematically identifying SNP-containing probes in GeneChip™ expression arrays. SK and LKV conceived the idea of automating the process of characterizing GeneChip™ probes with respect to SNPs, designed and implemented SNP-detection/data-analysis algorithms, AffyMAPSDetector tool. SK and LKV also performed the data analysis work with constant insights and critiques from JW. All authors read and approved the final manuscript.

## Supplementary Material

Additional file 1 **Complete SNP output file. **The listing of SNP-containing probes generated by AffyMAPSDetector for HG-U95Av2. Click here for file

Additional file 2 **Probes having SNP at mismatch location**. The listing of probes having a SNP exactly the 13^th ^position in the probe sequence. It also includes the probes that have SNPs at other position in addition to 13^th ^position. Click here for file

Additional file 3 **Probesets without SNPs**. The listing of all HG-U95Av2 probesets, none of whose probes were found to contain any documented SNPs. Click here for file

Additional file 4 **Genes with undefined LocusLink. **The listing of all HG-U95Av2 genes/probesets for which either LocusLink was not defined (typically genes sourced from TIGR) or AffyMAPSDetector couldn't disambiguate the given LocusLink information. Click here for file

Additional file 5 **HG-U95Av2 genes mRNA sequence. **The listing of HG-U95Av2 genes mRNA sequence extracted by AffyMAPSDetector from NCBI nucleotide database. Since size of this file is more than 80 mega bytes, only a truncated version of this file is provided here (less than 10 mega bytes – BMC upper limit). However, complete file can be obtained from the project home page. Click here for file

Additional file 6 **Additional SNP information for Probes having SNPs. **This file contains additional SNP information, extracted by AffyMAPSDetector from NCBI dbSNP, for probe having SNPs. Click here for file

Additional file 7 **AffyMAPSDetector execution log. **This file contains useful information logged by AffyMAPSDetector during HG-U95Av2 run. Click here for file

Additional file 8 **Behavior of SNP-containing probes with respect to PM and MM binding efficiencies. **This file presents examples of SNP-containing probes that affect PM and MM binding efficiencies in a tabular form with hyperlinks to dChip graphical images. Due to the large volume of data and size of the image files, this file is one of two parts. Each file, 8 and 9, unzips into its respective folder." Underneath each folder, you would see "effectOfSNPsOnExpression.html" file and "dChipGraphics" folder containing 71 image files in .emf format in each folder. For hyperlinks to work properly in "effectOfSNPsOnExpression.html" file, make sure that all image files reside together underneath one "dChipGraphics" folder (a total of 142 .emf files). Click here for file

Additional file 9 **Behavior of SNP-containing probes with respect to PM and MM binding efficiencies. **This file presents examples of SNP-containing probes that affect PM and MM binding efficiencies in a tabular form with hyperlinks to dChip graphical images. Click here for file

Additional file 10 **Example of probes affecting probeset detection calls. **This file contains some examples of SNP-containing probes that affect probeset detection call. Click here for file

Additional file 11 **SNP-containing probes' PM/MM ratio data file for expression genotype. **An instance of a PM/MM ratio data file from adenocarcinoma positive sample-replicates that can be loaded into the data visualization utility for expression signature pattern analysis. Click here for file

Additional file 12 **AffyMAPSDetector v1 distribution package (compiled code). **AffyMAPSDetector v1 distribution package (compiled code). Click here for file

Additional file 13 **AffyMAPSDetector v1 source code. **AffyMAPSDetector source code. Click here for file

Additional file 14 **Intensity distribution profiles' confirmation. **The set of files in this archive contain data analysis experiment results on lung adenocarcinoma dataset to confirm repeatability of intensity distribution patterns differences between probes with SNPs and probes without SNPs. Click here for file
