# Supplementary material for: AffyMAPSDetector: a software tool to characterize Affymetrix GeneChip™ expression arrays with respect to SNPs
Source: BMC Bioinformatics. 2007 Jul 30;8:276. doi: 10.1186/1471-2105-8-276 (PMC1959249; doi:10.1186/1471-2105-8-276)
Supplement: Additional file 8 — Behavior of SNP-containing probes with respect to PM and MM binding efficiencies. This file presents examples of SNP-containing probes that affect PM and MM binding efficiencies in a tabular form with hyperlinks to dChip graphical images. Due to the large volume of data and size of the image files, this file is one of two parts. Each file, 8 and 9, unzips into its respective folder." Underneath each folder, you would see "effectOfSNPsOnExpression.html" file and "dChipGraphics" folder containing 71 image files in .emf format in each folder. For hyperlinks to work properly in "effectOfSNPsOnExpression.html" file, make sure that all image files reside together underneath one "dChipGraphics" folder (a total of 142 .emf files). [file 1471-2105-8-276-S8.zip › file8a/file8a/effectOfSNPsOnExpression.html]

Effect of SNPs on Gene Expression


This table contains the examples of probes for which binding efficiencies of
PM and MM differ from sample to sample due to presence of one or
more SNPs. For each column the interpretation of the data is given below:

- Gene\_ID [Probeset\_ID(Probe)]: A SNP containing probe e.g. AF000152 [41202\_s\_at(166, 37)]  
  AF000152 - The NCBI gene identifier to which this probe belongs to.  
  41202\_s\_at - The probe-set id to which this probe belongs to.  
  (166,37) - Probe's (x,y) coordinates as laid out on the
  GeneChipTM(see chip definition file for details).  
  *Note:* Data in each cell under this column is hyperlinked to the dChip graphics
  of the probe-set (41202\_s\_at in this case) for all 190 samples. For each probe in a probe-set,
  the dChip graphics compares a samples' binding efficiency to PM and MM probes. All 190
  samples' PM and MM responses are arranged in grid (14 rows, 14 columns) of plots.
- dChip Graphics Probe Index: Position of the probe (starting at index 0 from
  left to right) in dChip graphics.  
   e.g. index 8 implies 9th probe in the
  probe-set "41202\_s\_at".
- Min Ratio (Best MM>PM): Representative sample showing higher binding
  to MM probe than PM probe, e.g., AD351T1\_A127\_3(CL2001032113AA) (0.73) [4, 5]  
  AD351T1\_A127\_3 - Sample identifier as defined in Lung Adenocarcinoma Dataset.  
  CL2001032113AA - Experiment identifier as defined in Lung Adenocarcinoma Dataset.  
  (0.73) - PM/MM expression ratio.  
  [4, 5] - Grid coordinates of "PM vs MM binding efficiency plot" in "CL2001032113AA"
  experiment for sample "AD351T1\_A127\_3" with respect to probe-set "41202\_s\_at". In this
  plot, 9th probe (at index 8) distinctly shows better binding to MM than PM.
- Max Ratio (Best PM>MM): Representative sample showing higher binding
  to PM probe than MM probe, e.g., AD375T1\_A115\_3(CL2001032322AA) (1.63) [8, 7]  
  AD375T1\_A115\_3 - Sample identifier as defined in Lung Adenocarcinoma Dataset.  
  CL2001032322AA - Experiment identifier as defined in Lung Adenocarcinoma Dataset.  
  (1.63) - PM/MM expression ratio.  
  [8, 7] - Grid coordinates in dChip graphics.
- PM Nearly Equal to MM: Representative sample showing relatively equal amount
  of binding to PM and MM probes, e.g., AD115T2\_A245\_10(CL2001032107AA) (1.0)[3, 14]  
  AD115T2\_A245\_10 - Sample identifier as defined in Lung Adenocarcinoma Dataset.  
  CL2001032107AA - Experiment identifier as defined in Lung Adenocarcinoma Dataset.  
  (1.0) - PM/MM expression ratio.  
  [3, 14] - Grid coordinates in dChip graphics.

|  |  |  |  |  |
| --- | --- | --- | --- | --- |
| **Gene\_ID [Probeset\_ID (Probe) ]** | **dChip Graphics Probe Index** | **Min Ratio (Best MM > PM)** | **Max Ratio (Best PM > MM)** | **PM Nearly Equal to MM** |
| AF000152 [41202\_s\_at(166, 37) ] | 8 | AD351T1\_A127\_3(CL2001032113AA) (0.73) [4, 5] | AD375T1\_A115\_3(CL2001032322AA) (1.63) [8, 7] | AD115T2\_A245\_10(CL2001032107AA) (1.0)[3, 14] |
| AF004876 [35326\_at(450, 101) ] | 11 | AD131T1\_A200\_8(CL2001032731AA) (0.68) [11, 10] | AD187T1\_A11\_1(CL2001032130AA) (1.53) [4, 11] | AD340T1\_A122\_3(CL2001031634AA) (1.0)[2, 2] |
| AF055008 [41198\_at(150, 581) ] | 14 | AD338T1\_A121\_3(CL2001032115AA) (0.67) [4, 7] | AD262T1\_A339\_6(CL2001031608AA) (1.28) [1, 1] | AD301T1\_A265\_10(CL2001032005AA) (1.0)[2, 6] |
| D14520 [37926\_at(348, 345) ] | 6 | AD163T3\_A205\_8(CL2001032833AA) (0.71) [12, 9] | ADA7T1\_A388\_7(CL2001031612AA) (1.89) [1, 3] | AD313T1\_A268\_10(CL2001031628AA) (1.0)[1, 11] |
| D29963 [36644\_at(330, 227) ] | 15 | AD334T1 \_A221\_8(CL2001032707AA) (0.52) [11, 1] | AD260T1\_A180\_4(CL2001031613AA) (1.68) [1, 4] | AD114T2\_A10\_1(CL2001032019AA) (1.0)[2, 14] |
| D38255 [37355\_at(514, 75) ] | 14 | AD366T1\_A109\_3(CL2001032116AA) (0.8) [4, 8] | AD218T1\_A147\_4(CL2001032310AA) (1.69) [7, 11] | ADA3T1\_A385\_7(CL2001031611AA) (1.0)[1, 2] |
| D50930 [40143\_at(126, 493) ] | 11 | AD351T1\_A127\_3(CL2001032113AA) (0.69) [4, 5] | AD361T1\_A177\_4(CL2001032134AA) (1.52) [4, 14] | AD350T1\_A126\_3(CL2001032111AA) (1.0)[4, 4] |
| L07597 [1127\_at(525, 21) ] | 9 | AD269T1\_A94\_3(CL2001031635AA) (0.57) [2, 3] | AD308T1\_A241\_8(CL2001032706AA) (6.08) [10, 14] | AD267T1\_A91\_3(CL2001032012AA) (1.0)[2, 10] |
| M20867 [37341\_at(372, 431) ] | 8 | ADA1T1\_A383\_7(CL2001042506AA) (0.74) [14, 8] | AD347T1\_A125\_3(CL2001040314AA) (1.31) [14, 1] | AD383T1\_A118\_3(CL2001031631AA) (1.0)[1, 14] |
| M98539 [216\_at(418, 415) ] | 13 | AD170T1\_A251\_10(CL2001032640AA) (0.29) [10, 5] | AD370T1\_A112\_3(CL2001032015AA) (2.7) [2, 12] | AD122T3\_A197\_8(CL2001032856AA) (1.0)[13, 8] |
| U03858 [1068\_g\_at(333, 387) ] | 8 | AD296T1\_A106\_3(CL2001032623AA) (0.75) [9, 10] | AD259T1\_A171\_4(CL2001032213AA) (1.19) [5, 11] | AD131T1\_A15\_1(CL2001031622AA) (1.0)[1, 8] |
| U90313 [824\_at(131, 553) ] | 10 | AD131T1\_A200\_8(CL2001032731AA) (0.74) [11, 10] | AD375T1\_A115\_3(CL2001032322AA) (1.53) [8, 7] | AD131T1\_A15\_1(CL2001031622AA) (1.0)[1, 8] |
| X02162 [39106\_at(80, 435) ] | 0 | AD338T1\_A130\_3(CL2001032244AA) (0.6) [6, 9] | AD360T2\_A406\_7(CL2001032036AA) (1.61) [3, 10] | AD131T1\_A15\_1(CL2001031622AA) (1.0)[1, 8] |
| X56681 [41484\_r\_at(623, 521) ] | 8 | AD285T2\_A263\_10(CL2001032717AA) (0.65) [11, 7] | AD266T1\_A90\_3(CL2001032013AA) (1.51) [2, 11] | AD177T1\_A21\_1(CL2001032601AA) (1.0)[8, 9] |
| X80822 [33614\_at(112, 361) ] | 15 | AD294T2\_A191\_4(CL2001032634AA) (0.61) [10, 1] | AD353T1\_A129\_3(CL2001032857AA) (1.56) [13, 9] | AD341T1\_A132\_3(CL2001031629AA) (1.0)[1, 12] |
| X96484 [40234\_at(17, 579) ] | 2 | AD258T1\_A187\_4(CL2001032615AA) (0.68) [9, 6] | AD259T1\_A171\_4(CL2001032213AA) (1.68) [5, 11] | AD158T2\_A17\_1(CL2001032235AA) (1.0)[6, 5] |
| AB000712 [35276\_at(140, 243) ] | 6 | AD120T1\_A226\_8(CL2001032739AA) (0.63) [12, 3] | AD340T1\_A122\_3(CL2001031634AA) (1.67) [2, 2] | AD383T1\_A118\_3(CL2001031631AA) (1.0)[1, 14] |
| AB000712 [35276\_at(296, 43) ] | 7 | AD360T1\_A176\_4(CL2001032639AA) (0.67) [10, 4] | AD340T1\_A131\_3(CL2001032319AA) (2.06) [8, 5] | AD266T1\_A90\_3(CL2001032013AA) (1.0)[2, 11] |
| D63878 [40281\_at(250, 425) ] | 10 | AD179T1\_A214\_8(CL2001032734AA) (0.65) [11, 12] | AD374T1\_A114\_3(CL2001032248AA) (2.22) [6, 12] | AD384T2 \_A288\_10(CL2001032105AA) (1.0)[3, 13] |
| D63878 [40281\_at(329, 425) ] | 11 | AD383T2\_A119\_3(CL2001032606AA) (0.73) [8, 14] | AD299T2\_A236\_8(CL2001032837AA) (1.43) [12, 11] | AD241T1\_A160\_4(CL2001032136AA) (1.0)[5, 2] |
| L13210 [37754\_at(191, 259) ] | 12 | AD131T1\_A200\_8(CL2001032731AA) (0.61) [11, 10] | AD353T1\_A129\_3(CL2001032857AA) (2.55) [13, 9] | AD301T1\_A237\_8(CL2001032741AA) (1.05)[12, 4] |
| L13210 [37754\_at(211, 483) ] | 6 | AD131T1\_A200\_8(CL2001032731AA) (0.32) [11, 10] | AD353T1\_A129\_3(CL2001032857AA) (1.68) [13, 9] | AD276T1\_A96\_3(CL2001032016AA) (1.0)[2, 13] |
| L13210 [37754\_at(543, 379) ] | 4 | AD131T1\_A200\_8(CL2001032731AA) (0.75) [11, 10] | AD353T1\_A129\_3(CL2001032857AA) (3.1) [13, 9] | AD299T1\_A235\_8(CL2001032742AA) (1.02)[12, 5] |
| AL080137 [32020\_at(133, 117) ] | 1 | AD255T1\_A169\_4(CL2001032135AA) (0.35) [5, 1] | AD352T1\_A128\_3(CL2001031632AA) (0.95) [2, 1] | Null |
| U15131 [37746\_r\_at(218, 55) ] | 11 | AD285T2\_A263\_10(CL2001032717AA) (0.39) [11, 7] | AD299T1\_A235\_8(CL2001032742AA) (0.96) [12, 5] | Null |
| U15131 [37746\_r\_at(386, 207) ] | 8 | AD285T2\_A263\_10(CL2001032717AA) (0.5) [11, 7] | AD261T1\_A173\_4(CL2001032214AA) (0.94) [5, 12] | Null |
| U15131 [37746\_r\_at(69, 185) ] | 14 | AD178T3\_A254\_10(CL2001032002AA) (0.72) [2, 5] | AD234T1\_A155\_4(CL2001032308AA) (1.02) [7, 9] | AD234T1\_A155\_4(CL2001032308AA) (1.02)[7, 9] |
| U15131 [37746\_r\_at(75, 333) ] | 3 | AD269T1\_A94\_3(CL2001031635AA) (0.76) [2, 3] | AD383T2\_A119\_3(CL2001032606AA) (1.19) [8, 14] | AD262T1\_A339\_6(CL2001031608AA) (1.0)[1, 1] |
| AB000095 [33448\_at(124, 573) ] | 8 | AD338T1\_A121\_3(CL2001032115AA) (0.94) [4, 7] | AD336T1\_A223\_8(CL2001041708AA) (1.59) [14, 2] | AD341T1\_A123\_3(CL2001032321AA) (1.04)[8, 6] |
| AB011076 [31393\_r\_at(606, 553) ] | 13 | AD173T1a\_A23\_1(CL2001032604AA) (0.68) [8, 12] | AD164T2\_A208\_8(CL2001032736AA) (1.69) [11, 14] | AD178T3\_A254\_10(CL2001032002AA) (1.0)[2, 5] |
| AB015202 [41602\_at(584, 89) ] | 7 | AD334T1 \_A221\_8(CL2001032707AA) (0.81) [11, 1] | AD260T1\_A180\_4(CL2001031613AA) (1.74) [1, 4] | AD167T1 \_A210\_8(CL2001032841AA) (1.0)[13, 1] |
| AB029343 [37424\_at(412, 149) ] | 5 | AD299T2\_A236\_8(CL2001032837AA) (0.52) [12, 11] | AD260T1\_A180\_4(CL2001031613AA) (1.58) [1, 4] | AD130T1\_A1\_1(CL2001032232AA) (1.0)[6, 2] |
| AF057160 [37303\_at(157, 27) ] | 11 | AD167T1 \_A210\_8(CL2001032841AA) (0.88) [13, 1] | AD366T1\_A109\_3(CL2001032116AA) (1.59) [4, 8] | AD362T1\_A282\_10(CL2001032602AA) (1.0)[8, 10] |
| AF109134 [40332\_at(390, 123) ] | 8 | AD336T1\_A223\_8(CL2001041708AA) (0.99) [14, 2] | AD255T1\_A178\_4(CL2001032227AA) (1.6) [5, 14] | AD338T1\_A121\_3(CL2001032115AA) (1.02)[4, 7] |
| J00153 [31525\_s\_at(149, 155) ] | 11 | AD360T1\_A176\_4(CL2001032639AA) (0.85) [10, 4] | AD350T1\_A126\_3(CL2001032111AA) (1.74) [4, 4] | AD384T2 \_A288\_10(CL2001032105AA) (1.0)[3, 13] |
| J04173 [41221\_at(90, 529) ] | 13 | AD202T2\_A139\_4(CL2001032612AA) (0.84) [9, 3] | AD374T1\_A114\_3(CL2001032248AA) (1.94) [6, 12] | AD301T1\_A237\_8(CL2001032741AA) (1.0)[12, 4] |
| S40719 [40185\_at(548, 33) ] | 15 | AD379T2\_A287\_10(CL2001032104AA) (0.89) [3, 12] | AD287T1\_A101\_3(CL2001031636AA) (1.64) [2, 4] | ADA7T1\_A388\_7(CL2001031612AA) (1.02)[1, 3] |
| S73885 [39638\_at(58, 567) ] | 15 | AD338T1\_A121\_3(CL2001032115AA) (0.87) [4, 7] | AD361T1\_A177\_4(CL2001032134AA) (1.66) [4, 14] | AD351T1\_A127\_3(CL2001032113AA) (1.0)[4, 5] |
| U09210 [31636\_s\_at(89, 47) ] | 6 | AD341T1\_A132\_3(CL2001031629AA) (0.85) [1, 12] | AD255T1\_A178\_4(CL2001032227AA) (1.43) [5, 14] | AD360T2\_A406\_7(CL2001032036AA) (1.01)[3, 10] |
| U89606 [35714\_at(187, 379) ] | 5 | AD131T1\_A200\_8(CL2001032731AA) (0.67) [11, 10] | AD374T1\_A114\_3(CL2001032248AA) (1.76) [6, 12] | AD311T1\_A266\_10(CL2001032257AA) (1.0)[7, 4] |
| Z96932 [32201\_at(314, 259) ] | 14 | AD187T2\_A233\_8(CL2001032743AA) (0.8) [12, 6] | AD360T1\_A176\_4(CL2001032639AA) (1.86) [10, 4] | AD383T1\_A118\_3(CL2001031631AA) (1.02)[1, 14] |
| U25849 [36611\_at(586, 133) ] | 12 | AD269T1\_A94\_3(CL2001031635AA) (0.9) [2, 3] | AD255T1\_A178\_4(CL2001032227AA) (2.02) [5, 14] | AD169T3\_A250\_10(CL2001032701AA) (1.02)[10, 10] |
| U25849 [36611\_at(587, 133) ] | 11 | AD269T1\_A94\_3(CL2001031635AA) (0.81) [2, 3] | AD353T1\_A129\_3(CL2001032857AA) (1.87) [13, 9] | AD111T2\_A8\_1(CL2001032233AA) (1.01)[6, 3] |
| AF040958 [39075\_at(457, 377) ] | 10 | AD183T1\_A6\_8(CL2001032840AA) (0.97) [12, 14] | ADA2T1\_A384\_7(CL2001032842AA) (1.54) [13, 2] | AD262T1\_A259\_10(CL2001032315AA) (1.02)[8, 2] |
| AF040958 [39075\_at(524, 417) ] | 9 | AD338T1\_A121\_3(CL2001032115AA) (0.69) [4, 7] | ADA2T1\_A384\_7(CL2001032842AA) (1.01) [13, 2] | AD370T1\_A112\_3(CL2001032015AA) (1.01)[2, 12] |
| D38498 [1875\_f\_at(499, 253) ] | 15 | AD249T1 \_A165\_4(CL2001032617AA) (0.81) [9, 8] | AD363T1\_A283\_10(CL2001032848AA) (1.87) [13, 7] | ADA19T1\_A393\_7(CL2001032307AA) (1.0)[7, 8] |
| M96326 [33963\_at(7, 343) ] | 8 | AD277T1\_A97\_3(CL2001032255AA) (0.99) [7, 3] | ADA7T1\_A388\_7(CL2001031612AA) (1.85) [1, 3] | AD283T1\_A99\_3(CL2001032254AA) (1.07)[7, 2] |
| M96326 [33963\_at(92, 181) ] | 9 | AD283T1\_A99\_3(CL2001032254AA) (0.99) [7, 2] | AD363T1\_A283\_10(CL2001032848AA) (4.26) [13, 7] | AD275T1\_A95\_3(CL2001032311AA) (1.0)[7, 12] |
| AF017307 [40445\_at(3, 35) ] | 2 | AD383T1\_A118\_3(CL2001031631AA) (0.98) [1, 14] | AD315T1\_A270\_10(CL2001041716AA) (2.12) [14, 5] | AD352T1\_A128\_3(CL2001031632AA) (1.0)[2, 1] |
| AF026292 [38720\_at(496, 251) ] | 13 | AD285T2\_A263\_10(CL2001032717AA) (0.95) [11, 7] | ADA16T2\_A391\_7(CL2001032149AA) (2.38) [5, 6] | AD208T1\_A143\_4(CL2001032229AA) (1.05)[6, 1] |
| D21261 [36678\_at(592, 7) ] | 7 | AD185T2\_A232\_8(CL2001032843AA) (0.73) [13, 3] | AD327T3\_A277\_10(CL2001032713AA) (2.29) [11, 4] | AD301T1\_A237\_8(CL2001032741AA) (1.0)[12, 4] |
| D63486 [41728\_at(259, 83) ] | 11 | AD115T2\_A245\_10(CL2001032107AA) (0.65) [3, 14] | ADA7T1\_A388\_7(CL2001031612AA) (2.38) [1, 3] | AD301T1\_A237\_8(CL2001032741AA) (1.0)[12, 4] |
| J03077 [36795\_at(47, 33) ] | 7 | ADA10T1\_A389\_7(CL2001032716AA) (0.72) [11, 6] | AD315T1\_A270\_10(CL2001041716AA) (2.1) [14, 5] | AD382T1\_A117\_3(CL2001040304AA) (1.01)[13, 11] |
| J03077 [36795\_at(638, 385) ] | 3 | AD115T2\_A245\_10(CL2001032107AA) (0.8) [3, 14] | AD374T1\_A114\_3(CL2001032248AA) (2.71) [6, 12] | AD384T2 \_A288\_10(CL2001032105AA) (1.01)[3, 13] |
| L09159 [37309\_at(258, 199) ] | 14 | AD131T1\_A200\_8(CL2001032731AA) (0.93) [11, 10] | AD163T3\_A205\_8(CL2001032833AA) (2.25) [12, 9] | AD362T1\_A282\_10(CL2001032602AA) (1.1)[8, 10] |
| L19686 [895\_at(252, 593) ] | 14 | ADA18T1\_A392\_7(CL2001032301AA) (0.71) [7, 6] | AD382T1\_A117\_3(CL2001040304AA) (2.25) [13, 11] | AD311T1\_A266\_10(CL2001032257AA) (1.0)[7, 4] |
| M16279 [41138\_at(176, 501) ] | 15 | AD186T1\_A27\_1(CL2001032125AA) (0.89) [4, 10] | AD123T2\_A198\_8(CL2001042502AA) (2.34) [14, 6] | AD173T1a\_A23\_1(CL2001032604AA) (1.07)[8, 12] |
| M64716 [31573\_at(292, 127) ] | 10 | AD203T2\_A141\_4(CL2001032605AA) (0.96) [8, 13] | AD315T1\_A270\_10(CL2001041716AA) (2.14) [14, 5] | AD334T1 \_A221\_8(CL2001032707AA) (1.0)[11, 1] |
| M92843 [40448\_at(535, 95) ] | 8 | AD157T1\_A246\_10(CL2001032250AA) (0.93) [6, 14] | AD315T1\_A270\_10(CL2001041716AA) (2.3) [14, 5] | AD338T1\_A121\_3(CL2001032115AA) (1.01)[4, 7] |
| U12472 [33396\_at(338, 133) ] | 3 | AD336T1\_A223\_8(CL2001041708AA) (0.69) [14, 2] | AD183T1\_A6\_8(CL2001032840AA) (4.17) [12, 14] | AD313T1\_A268\_10(CL2001031628AA) (1.08)[1, 11] |
| U37690 [503\_at(621, 245) ] | 9 | AD177T1\_A21\_1(CL2001032601AA) (0.81) [8, 9] | ADA31\_A289\_10(CL2001032259AA) (2.03) [7, 5] | AD203T2\_A141\_4(CL2001032605AA) (1.0)[8, 13] |
| X55954 [32395\_r\_at(21, 455) ] | 3 | AD301T1\_A237\_8(CL2001032741AA) (0.99) [12, 4] | AD353T1\_A129\_3(CL2001032857AA) (2.31) [13, 9] | AD334T1 \_A221\_8(CL2001032707AA) (1.07)[11, 1] |
| U07132 [519\_g\_at(318, 355) ] | 10 | AD159T1\_A229\_8(CL2001032839AA) (0.58) [12, 13] | AD370T1\_A112\_3(CL2001032015AA) (1.1) [2, 12] | AD374T1\_A114\_3(CL2001032248AA) (1.0)[6, 12] |
| U07132 [519\_g\_at(626, 247) ] | 6 | AD299T1\_A235\_8(CL2001032742AA) (0.55) [12, 5] | AD309T1\_A242\_8(CL2001041711AA) (2.45) [14, 3] | AD338T1\_A121\_3(CL2001032115AA) (1.02)[4, 7] |
| X00457 [38833\_at(529, 25) ] | 7 | AD131T1\_A200\_8(CL2001032731AA) (0.69) [11, 10] | ADA31\_A289\_10(CL2001032259AA) (4.15) [7, 5] | AD335T2\_A281\_10(CL2001032008AA) (1.13)[2, 7] |
| X00457 [38833\_at(556, 171) ] | 15 | AD362T1\_A282\_10(CL2001032602AA) (0.94) [8, 10] | AD353T1\_A129\_3(CL2001032857AA) (2.77) [13, 9] | AD169T2\_A211\_8(CL2001032735AA) (1.02)[11, 13] |
| X00457 [38833\_at(611, 5) ] | 5 | AD131T1\_A200\_8(CL2001032731AA) (0.33) [11, 10] | ADA31\_A289\_10(CL2001032259AA) (1.28) [7, 5] | AD243T1\_A161\_4(CL2001032318AA) (1.01)[8, 4] |
| V00599 [151\_s\_at(622, 367) ] | 7 | AD338T1\_A121\_3(CL2001032115AA) (0.82) [4, 7] | AD360T2\_A406\_7(CL2001032036AA) (2.0) [3, 10] | AD187T2\_A233\_8(CL2001032743AA) (1.02)[12, 6] |
| V00599 [151\_s\_at(638, 303) ] | 8 | AD338T1\_A121\_3(CL2001032115AA) (0.57) [4, 7] | AD335T2\_A281\_10(CL2001032008AA) (2.44) [2, 7] | AD347T1\_A125\_3(CL2001040314AA) (1.0)[14, 1] |
| M63138 [239\_at(200, 625) ] | 13 | AD131T1\_A200\_8(CL2001032731AA) (0.62) [11, 10] | AD374T1\_A114\_3(CL2001032248AA) (2.67) [6, 12] | AD268T2\_A189\_4(CL2001032633AA) (1.0)[9, 14] |
| M63138 [239\_at(607, 307) ] | 6 | AD309T1\_A242\_8(CL2001041711AA) (0.76) [14, 3] | AD262T1\_A259\_10(CL2001032315AA) (2.91) [8, 2] | Null |
| AB009462 [31814\_i\_at(341, 217) ] | 11 | AD178T3\_A254\_10(CL2001032002AA) (0.26) [2, 5] | AD347T1\_A134\_3(CL2001032243AA) (0.52) [6, 8] | Null |
| AB009462 [31814\_i\_at(38, 505) ] | 10 | AD350T1\_A126\_3(CL2001032111AA) (0.23) [4, 4] | AD347T1\_A134\_3(CL2001032243AA) (0.78) [6, 8] | Null |
| AB028944 [41492\_r\_at(601, 343) ] | 2 | AD338T1\_A121\_3(CL2001032115AA) (0.36) [4, 7] | AD183T1\_A215\_1(CL2001032117AA) (0.76) [4, 9] | Null |
| AF007134 [41279\_f\_at(230, 601) ] | 11 | AD350T1\_A126\_3(CL2001032111AA) (0.31) [4, 4] | AD276T1\_A96\_3(CL2001032016AA) (0.91) [2, 13] | Null |
| AF035279 [41677\_at(9, 343) ] | 2 | AD172T4\_A252\_10(CL2001040307AA) (0.47) [13, 14] | AD335T1\_A222\_8(CL2001032737AA) (0.79) [12, 1] | Null |
| AF068006 [37790\_at(11, 421) ] | 5 | AD186T1\_A27\_1(CL2001032125AA) (0.43) [4, 10] | AD341T1\_A132\_3(CL2001031629AA) (0.67) [1, 12] | Null |
| AF070530 [35230\_at(444, 297) ] | 6 | AD267T1\_A91\_3(CL2001032012AA) (0.42) [2, 10] | AD261T1\_A173\_4(CL2001032214AA) (0.73) [5, 12] | Null |
| J00140 [37913\_at(91, 567) ] | 5 | AD302T3\_A238\_8(CL2001032838AA) (0.56) [12, 12] | AD338T1\_A130\_3(CL2001032244AA) (0.96) [6, 9] | Null |
| L04490 [36205\_at(569, 3) ] | 2 | AD269T1\_A94\_3(CL2001031635AA) (0.29) [2, 3] | AD336T1\_A223\_8(CL2001041708AA) (0.65) [14, 2] | Null |
| L18983 [917\_g\_at(179, 545) ] | 8 | AD163T3\_A205\_8(CL2001032833AA) (0.55) [12, 9] | AD252T1\_A167\_4(CL2001032030AA) (0.95) [3, 6] | Null |
| L19559 [37228\_at(481, 41) ] | 13 | AD338T1\_A121\_3(CL2001032115AA) (0.62) [4, 7] | AD301T1\_A265\_10(CL2001032005AA) (0.89) [2, 6] | Null |
| L37127 [1486\_at(137, 419) ] | 15 | AD179T2 \_A255\_10(CL2001032249AA) (0.51) [6, 13] | AD203T1\_A140\_4(CL2001032025AA) (0.82) [3, 2] | Null |
| L37127 [1486\_at(626, 489) ] | 11 | AD122T3\_A197\_8(CL2001032856AA) (0.49) [13, 8] | AD203T2\_A141\_4(CL2001032605AA) (0.88) [8, 13] | Null |
| L76191 [1100\_at(533, 289) ] | 6 | AD261T1\_A173\_4(CL2001032214AA) (0.3) [5, 12] | AD320T1\_A272\_10(CL2001040305AA) (0.66) [13, 12] | Null |
| M13194 [1878\_g\_at(451, 259) ] | 13 | ADA16T2\_A391\_7(CL2001032149AA) (0.31) [5, 6] | AD375T1\_A115\_3(CL2001032322AA) (0.58) [8, 7] | Null |
| M26062 [1365\_at(436, 415) ] | 7 | AD120T1\_A226\_8(CL2001032739AA) (0.51) [12, 3] | AD366T1\_A109\_3(CL2001032116AA) (0.92) [4, 8] | Null |
| M35011 [2058\_s\_at(221, 553) ] | 7 | ADA19T1\_A393\_7(CL2001032307AA) (0.29) [7, 8] | AD230T1\_A153\_4(CL2001032142AA) (0.71) [5, 5] | Null |
| M65199 [1092\_at(300, 407) ] | 11 | AD259T1\_A171\_4(CL2001032213AA) (0.29) [5, 11] | AD267T1\_A91\_3(CL2001032012AA) (0.58) [2, 10] | Null |
| U05875 [41140\_at(472, 375) ] | 2 | AD269T1\_A94\_3(CL2001031635AA) (0.15) [2, 3] | AD338T1\_A121\_3(CL2001032115AA) (0.56) [4, 7] | Null |
| U09607 [1547\_at(441, 233) ] | 7 | AD253T1\_A168\_4(CL2001031616AA) (0.46) [1, 6] | ADA2T1\_A384\_7(CL2001032842AA) (0.78) [13, 2] | Null |
| U15131 [37746\_r\_at(205, 207) ] | 4 | AD169T2\_A211\_8(CL2001032735AA) (0.37) [11, 13] | AD230T1\_A153\_4(CL2001032142AA) (0.76) [5, 5] | Null |
| U38276 [35666\_at(293, 283) ] | 4 | AD294T1\_A104\_3(CL2001032114AA) (0.56) [4, 6] | AD228T2\_A152\_4(CL2001032312AA) (0.93) [7, 13] | Null |
| U43923 [508\_at(365, 327) ] | 14 | AD353T1\_A129\_3(CL2001032857AA) (0.4) [13, 9] | AD183T1\_A215\_1(CL2001032117AA) (0.92) [4, 9] | Null |
| U45974 [39323\_at(38, 167) ] | 14 | AD131T1\_A200\_8(CL2001032731AA) (0.54) [11, 10] | AD383T1\_A118\_3(CL2001031631AA) (0.98) [1, 14] | Null |
| U58917 [36229\_at(475, 183) ] | 5 | AD301T1\_A237\_8(CL2001032741AA) (0.42) [12, 4] | AD336T1\_A223\_8(CL2001041708AA) (0.7) [14, 2] | Null |
| U59423 [1325\_at(142, 597) ] | 12 | AD301T1\_A237\_8(CL2001032741AA) (0.27) [12, 4] | AD283T1\_A99\_3(CL2001032254AA) (0.71) [7, 2] | Null |
| U62437 [34154\_at(30, 181) ] | 10 | AD163T1\_A203\_8(CL2001041714AA) (0.45) [14, 4] | AD283T1\_A99\_3(CL2001032254AA) (0.91) [7, 2] | Null |
| U66619 [456\_at(546, 591) ] | 15 | AD311T2\_A267\_10(CL2001032103AA) (0.21) [3, 11] | AD252T1\_A167\_4(CL2001032030AA) (0.61) [3, 6] | Null |
| U84763 [34421\_g\_at(427, 29) ] | 9 | AD302T3\_A238\_8(CL2001032838AA) (0.43) [12, 12] | AD375T1\_A115\_3(CL2001032322AA) (0.93) [8, 7] | Null |
| U90313 [824\_at(539, 151) ] | 11 | AD163T1\_A203\_8(CL2001041714AA) (0.15) [14, 4] | AD169T3\_A250\_10(CL2001032701AA) (0.59) [10, 10] | Null |
| U96721 [38467\_at(347, 181) ] | 5 | AD356T1\_A175\_4(CL2001032034AA) (0.44) [3, 9] | AD169T2\_A211\_8(CL2001032735AA) (0.75) [11, 13] | Null |
| X06882 [36661\_s\_at(626, 233) ] | 14 | AD169T3\_A250\_10(CL2001032701AA) (0.43) [10, 10] | ADA4T1\_A386\_7(CL2001032155AA) (0.89) [5, 7] | Null |
| X51362 [40371\_at(195, 239) ] | 5 | AD302T3\_A238\_8(CL2001032838AA) (0.43) [12, 12] | AD318T3\_A107\_3(CL2001040306AA) (0.66) [13, 13] | Null |
| X96484 [40234\_at(67, 567) ] | 13 | AD178T2\_A22\_1(CL2001032234AA) (0.24) [6, 4] | AD243T2\_A257\_10(CL2001032108AA) (0.46) [4, 1] | Null |
| X97267 [32070\_at(368, 63) ] | 12 | AD315T1\_A270\_10(CL2001041716AA) (0.48) [14, 5] | AD119T3\_A195\_8(CL2001032845AA) (0.96) [13, 5] | Null |
| X97548 [33425\_at(384, 375) ] | 13 | AD269T1\_A94\_3(CL2001031635AA) (0.44) [2, 3] | AD183T1\_A6\_8(CL2001032840AA) (0.78) [12, 14] | Null |
| Z69043 [38635\_at(417, 281) ] | 1 | AD136T2\_A201\_8(CL2001032729AA) (0.43) [11, 8] | ADA31\_A289\_10(CL2001032259AA) (0.88) [7, 5] | Null |
| AB007867 [33783\_at(140, 295) ] | 11 | AD120T1\_A226\_8(CL2001032739AA) (0.52) [12, 3] | AD379T2\_A287\_10(CL2001032104AA) (1.02) [3, 12] | AD379T2\_A287\_10(CL2001032104AA) (1.02)[3, 12] |
| AB007867 [33783\_at(25, 171) ] | 12 | ADA1T1\_A383\_7(CL2001042506AA) (0.42) [14, 8] | AD275T1\_A95\_3(CL2001032311AA) (0.68) [7, 12] | Null |
| AB000584 [1890\_at(333, 217) ] | 14 | AD269T1\_A94\_3(CL2001031635AA) (0.4) [2, 3] | AD167T2 \_A249\_10(CL2001032858AA) (1.47) [13, 10] | AD213T1\_A146\_4(CL2001032309AA) (1.01)[7, 10] |
| AB002308 [35313\_at(68, 271) ] | 15 | AD173T1a\_A23\_1(CL2001032604AA) (0.67) [8, 12] | AD374T1\_A114\_3(CL2001032248AA) (1.09) [6, 12] | AD252T1\_A167\_4(CL2001032030AA) (1.0)[3, 6] |
| AB009398 [32211\_at(365, 597) ] | 0 | AD186T1\_A27\_1(CL2001032125AA) (0.65) [4, 10] | AD375T1\_A115\_3(CL2001032322AA) (1.04) [8, 7] | AD315T1\_A270\_10(CL2001041716AA) (1.0)[14, 5] |
| AB018306 [34832\_s\_at(74, 183) ] | 11 | AD186T1\_A27\_1(CL2001032125AA) (0.64) [4, 10] | AD346T1\_A133\_3(CL2001032324AA) (1.04) [8, 8] | AD295T1\_A105\_3(CL2001032252AA) (1.0)[7, 1] |
| AB028972 [41268\_g\_at(287, 269) ] | 14 | AD186T1\_A27\_1(CL2001032125AA) (0.51) [4, 10] | AD170T1\_A251\_10(CL2001032640AA) (1.08) [10, 5] | AD201T1\_A138\_4(CL2001032141AA) (1.0)[5, 4] |
| AF016898 [39942\_at(54, 589) ] | 9 | AD179T1\_A214\_8(CL2001032734AA) (0.55) [11, 12] | AD258T1\_A170\_4(CL2001032646AA) (1.25) [10, 8] | AD269T1\_A94\_3(CL2001031635AA) (1.02)[2, 3] |
| AF026031 [34345\_at(350, 399) ] | 14 | AD226T2\_A151\_4(CL2001031618AA) (0.74) [1, 7] | AD277T1\_A97\_3(CL2001032255AA) (1.16) [7, 3] | ADA7T1\_A388\_7(CL2001031612AA) (1.0)[1, 3] |
| AF027302 [39141\_at(2, 163) ] | 3 | AD350T1\_A126\_3(CL2001032111AA) (0.51) [4, 4] | AD335T2\_A281\_10(CL2001032008AA) (1.03) [2, 7] | AD352T1\_A128\_3(CL2001031632AA) (1.01)[2, 1] |
| AF044896 [41409\_at(429, 149) ] | 6 | AD111T2\_A8\_1(CL2001032233AA) (0.5) [6, 3] | AD269T1\_A94\_3(CL2001031635AA) (1.34) [2, 3] | AD346T1\_A124\_3(CL2001032011AA) (1.0)[2, 9] |
| AF044896 [41409\_at(458, 281) ] | 5 | AD268T2\_A189\_4(CL2001032633AA) (0.43) [9, 14] | ADA1T1\_A383\_7(CL2001042506AA) (1.14) [14, 8] | AD269T1\_A94\_3(CL2001031635AA) (1.13)[2, 3] |
| AF053944 [39069\_at(162, 453) ] | 12 | AD186T1\_A27\_1(CL2001032125AA) (0.58) [4, 10] | AD375T1\_A115\_3(CL2001032322AA) (1.12) [8, 7] | AD259T1\_A171\_4(CL2001032213AA) (1.01)[5, 11] |
| AF067224 [33709\_at(103, 479) ] | 6 | AD350T1\_A135\_3(CL2001032635AA) (0.11) [10, 2] | AD379T2\_A287\_10(CL2001032104AA) (1.16) [3, 12] | AD379T2\_A287\_10(CL2001032104AA) (1.16)[3, 12] |
| AF067575 [41222\_at(328, 225) ] | 10 | AD302T4\_A239\_8(CL2001032844AA) (0.59) [13, 4] | AD225T1\_A150\_4(CL2001032647AA) (1.25) [10, 9] | ADA4T1\_A386\_7(CL2001032155AA) (1.0)[5, 7] |
| AJ002308 [34885\_at(281, 289) ] | 13 | AD131T1\_A200\_8(CL2001032731AA) (0.4) [11, 10] | ADA4T1\_A386\_7(CL2001032155AA) (1.29) [5, 7] | AD252T1\_A167\_4(CL2001032030AA) (1.0)[3, 6] |
| AL050284 [34151\_at(583, 17) ] | 8 | AD179T1\_A214\_8(CL2001032734AA) (0.48) [11, 12] | AD360T2\_A406\_7(CL2001032036AA) (1.02) [3, 10] | AD338T1\_A121\_3(CL2001032115AA) (1.0)[4, 7] |
| D42040 [36208\_at(619, 111) ] | 3 | AD169T2\_A211\_8(CL2001032735AA) (0.68) [11, 13] | AD315T1\_A270\_10(CL2001041716AA) (1.2) [14, 5] | AD335T2\_A281\_10(CL2001032008AA) (1.0)[2, 7] |
| D87953 [36933\_at(237, 619) ] | 8 | AD115T2\_A245\_10(CL2001032107AA) (0.29) [3, 14] | ADA4T1\_A386\_7(CL2001032155AA) (1.46) [5, 7] | AD167T1 \_A210\_8(CL2001032841AA) (1.01)[13, 1] |
| J00287 [36642\_at(92, 631) ] | 11 | AD127T1\_A14\_1(CL2001032241AA) (0.44) [6, 6] | ADA15T1\_A390\_7(CL2001032306AA) (0.91) [7, 7] | Null |
| J02871 [1667\_s\_at(360, 477) ] | 13 | AD130T1\_A1\_1(CL2001032232AA) (0.44) [6, 2] | AD327T1\_A276\_10(CL2001031625AA) (1.18) [1, 9] | AD327T1\_A276\_10(CL2001031625AA) (1.18)[1, 9] |
| L37033 [40850\_at(406, 181) ] | 13 | AD159T1\_A229\_8(CL2001032839AA) (0.59) [12, 13] | ADA31\_A289\_10(CL2001032259AA) (1.14) [7, 5] | ADA31\_A289\_10(CL2001032259AA) (1.14)[7, 5] |
| M21302 [36734\_at(163, 19) ] | 5 | AD239T1\_A158\_4(CL2001032132AA) (0.44) [4, 12] | AD295T1\_A105\_3(CL2001032252AA) (1.27) [7, 1] | AD295T1\_A105\_3(CL2001032252AA) (1.27)[7, 1] |
| M21302 [36734\_at(306, 103) ] | 1 | ADA5T1\_A387\_7(CL2001042505AA) (0.65) [14, 7] | AD295T1\_A105\_3(CL2001032252AA) (1.24) [7, 1] | AD323T1\_A273\_10(CL2001032110AA) (1.0)[4, 3] |
| M21302 [36734\_at(91, 151) ] | 8 | AD212T1\_A145\_4(CL2001032138AA) (0.59) [5, 3] | AD295T1\_A105\_3(CL2001032252AA) (1.27) [7, 1] | AD323T1\_A273\_10(CL2001032110AA) (1.06)[4, 3] |
| M23410 [2047\_s\_at(240, 317) ] | 14 | AD269T1\_A94\_3(CL2001031635AA) (0.43) [2, 3] | AD341T1\_A123\_3(CL2001032321AA) (1.19) [8, 6] | AD201T1\_A138\_4(CL2001032141AA) (1.0)[5, 4] |
| M31523 [1374\_g\_at(196, 217) ] | 9 | AD188T1\_A216\_8(CL2001032733AA) (0.6) [11, 11] | AD277T1\_A97\_3(CL2001032255AA) (1.26) [7, 3] | AD352T1\_A128\_3(CL2001031632AA) (1.0)[2, 1] |
| M33146 [38700\_at(58, 563) ] | 6 | AD131T1\_A200\_8(CL2001032731AA) (0.65) [11, 10] | AD370T1\_A112\_3(CL2001032015AA) (1.77) [2, 12] | AD340T1\_A122\_3(CL2001031634AA) (1.0)[2, 2] |
| M64788 [1251\_g\_at(137, 605) ] | 12 | AD382T1\_A117\_3(CL2001040304AA) (0.44) [13, 11] | AD308T1\_A241\_8(CL2001032706AA) (1.04) [10, 14] | AD308T1\_A241\_8(CL2001032706AA) (1.04)[10, 14] |
| M73554 [2020\_at(370, 431) ] | 6 | AD332T1\_A220\_8(CL2001032747AA) (0.57) [12, 8] | AD162T2\_A230\_8(CL2001032702AA) (1.28) [10, 11] | AD164T1a\_A206\_8(CL2001032703AA) (1.01)[10, 12] |
| M75165 [32313\_at(100, 357) ] | 7 | AD338T1\_A121\_3(CL2001032115AA) (0.6) [4, 7] | AD375T1\_A115\_3(CL2001032322AA) (1.64) [8, 7] | AD221T1\_A148\_4(CL2001031615AA) (1.0)[1, 5] |
| M94345 [38391\_at(130, 403) ] | 13 | AD351T1\_A127\_3(CL2001032113AA) (0.56) [4, 5] | AD260T1\_A180\_4(CL2001031613AA) (1.14) [1, 4] | ADA1T1\_A383\_7(CL2001042506AA) (1.0)[14, 8] |
| U07418 [1850\_at(497, 151) ] | 2 | AD178T3\_A254\_10(CL2001032002AA) (0.51) [2, 5] | AD336T1\_A223\_8(CL2001041708AA) (1.23) [14, 2] | AD370T1\_A112\_3(CL2001032015AA) (1.09)[2, 12] |
| U19345 [33793\_at(286, 375) ] | 9 | ADA16T2\_A391\_7(CL2001032149AA) (0.52) [5, 6] | AD336T1\_A223\_8(CL2001041708AA) (1.09) [14, 2] | AD336T1\_A223\_8(CL2001041708AA) (1.09)[14, 2] |
| U56418 [32837\_at(146, 237) ] | 7 | AD379T2\_A287\_10(CL2001032104AA) (0.58) [3, 12] | AD268T2 \_A262\_10(CL2001032009AA) (1.5) [2, 8] | AD361T1\_A177\_4(CL2001032134AA) (1.01)[4, 14] |
| U58048 [41773\_at(182, 359) ] | 9 | AD268T2\_A189\_4(CL2001032633AA) (0.66) [9, 14] | ADA31\_A289\_10(CL2001032259AA) (1.23) [7, 5] | AD118T1\_A13\_1(CL2001032020AA) (1.0)[3, 1] |
| U58917 [36229\_at(545, 597) ] | 5 | AD276T2\_A190\_4(CL2001032133AA) (0.54) [4, 13] | AD374T1\_A114\_3(CL2001032248AA) (1.0) [6, 12] | AD374T1\_A114\_3(CL2001032248AA) (1.0)[6, 12] |
| U81787 [1018\_at(520, 121) ] | 9 | AD239T1\_A158\_4(CL2001032132AA) (0.59) [4, 12] | AD302T3\_A238\_8(CL2001032838AA) (1.18) [12, 12] | AD302T3\_A238\_8(CL2001032838AA) (1.18)[12, 12] |
| U83246 [40452\_at(218, 285) ] | 12 | ADA19T1\_A393\_7(CL2001032307AA) (0.55) [7, 8] | AD374T1\_A114\_3(CL2001032248AA) (1.19) [6, 12] | ADA5T1\_A387\_7(CL2001042505AA) (1.0)[14, 7] |
| U83246 [40452\_at(520, 79) ] | 11 | AD230T1\_A153\_4(CL2001032142AA) (0.52) [5, 5] | AD374T1\_A114\_3(CL2001032248AA) (1.27) [6, 12] | ADA7T1\_A388\_7(CL2001031612AA) (1.01)[1, 3] |
| X05299 [37931\_at(104, 279) ] | 3 | ADA1T1\_A383\_7(CL2001042506AA) (0.34) [14, 8] | AD350T1\_A126\_3(CL2001032111AA) (1.07) [4, 4] | AD350T1\_A126\_3(CL2001032111AA) (1.07)[4, 4] |
| X05332 [40794\_at(584, 435) ] | 9 | AD302T3\_A238\_8(CL2001032838AA) (0.56) [12, 12] | AD347T1\_A125\_3(CL2001040314AA) (1.03) [14, 1] | AD320T1\_A272\_10(CL2001040305AA) (1.01)[13, 12] |
| X13710 [37033\_s\_at(180, 201) ] | 2 | AD131T1\_A200\_8(CL2001032731AA) (0.42) [11, 10] | AD374T1\_A114\_3(CL2001032248AA) (1.24) [6, 12] | AD260T1\_A172\_4(CL2001032028AA) (1.0)[3, 5] |
| X61123 [37294\_at(557, 213) ] | 3 | AD172T4\_A252\_10(CL2001040307AA) (0.56) [13, 14] | AD368T2\_A285\_10(CL2001032314AA) (1.41) [8, 1] | AD299T1\_A235\_8(CL2001032742AA) (1.0)[12, 5] |
| X63564 [40791\_at(480, 451) ] | 8 | AD221T1\_A148\_4(CL2001031615AA) (0.8) [1, 5] | AD269T1\_A94\_3(CL2001031635AA) (1.2) [2, 3] | AD260T1\_A180\_4(CL2001031613AA) (1.0)[1, 4] |
| AF065388 [34775\_at(132, 561) ] | 14 | AD360T1\_A176\_4(CL2001032639AA) (0.59) [10, 4] | AD341T1\_A123\_3(CL2001032321AA) (2.72) [8, 6] | AD239T1\_A158\_4(CL2001032132AA) (1.0)[4, 12] |
| M21389 [613\_at(419, 191) ] | 13 | AD302T3\_A238\_8(CL2001032838AA) (0.57) [12, 12] | AD341T1\_A123\_3(CL2001032321AA) (2.02) [8, 6] | AD131T1\_A200\_8(CL2001032731AA) (1.0)[11, 10] |
| Y13710 [32128\_at(443, 215) ] | 6 | AD336T1\_A223\_8(CL2001041708AA) (0.48) [14, 2] | AD225T1\_A150\_4(CL2001032647AA) (3.46) [10, 9] | AD239T1\_A158\_4(CL2001032132AA) (1.0)[4, 12] |
| AB002321 [32592\_at(558, 191) ] | 12 | AD340T1\_A131\_3(CL2001032319AA) (0.74) [8, 5] | AD120T1\_A226\_8(CL2001032739AA) (2.63) [12, 3] | AD218T1\_A147\_4(CL2001032310AA) (1.0)[7, 11] |
| AF089750 [40635\_at(141, 545) ] | 11 | AD186T1\_A27\_1(CL2001032125AA) (0.59) [4, 10] | AD299T2\_A236\_8(CL2001032837AA) (2.12) [12, 11] | AD252T1\_A167\_4(CL2001032030AA) (1.0)[3, 6] |
| AF089750 [40635\_at(511, 133) ] | 3 | AD186T1\_A27\_1(CL2001032125AA) (0.63) [4, 10] | AD335T2\_A281\_10(CL2001032008AA) (2.05) [2, 7] | AD226T2\_A151\_4(CL2001031618AA) (1.0)[1, 7] |
| D63475 [39795\_at(506, 151) ] | 3 | AD338T1\_A130\_3(CL2001032244AA) (0.87) [6, 9] | ADA10T1\_A389\_7(CL2001032716AA) (3.95) [11, 6] | AD250T1\_A166\_4(CL2001032031AA) (1.07)[3, 7] |
| M60028 [36878\_f\_at(201, 627) ] | 4 | AD335T1\_A222\_8(CL2001032737AA) (0.71) [12, 1] | ADA31\_A289\_10(CL2001032259AA) (2.77) [7, 5] | AD341T1\_A132\_3(CL2001031629AA) (1.0)[1, 12] |
| M80482 [32001\_s\_at(57, 563) ] | 9 | AD366T1\_A109\_3(CL2001032116AA) (0.72) [4, 8] | AD308T1\_A241\_8(CL2001032706AA) (3.09) [10, 14] | AD268T2 \_A262\_10(CL2001032009AA) (1.0)[2, 8] |
| M81141 [36773\_f\_at(227, 47) ] | 8 | AD269T1\_A94\_3(CL2001031635AA) (0.64) [2, 3] | AD353T1\_A129\_3(CL2001032857AA) (2.32) [13, 9] | AD368T2\_A285\_10(CL2001032314AA) (1.0)[8, 1] |
| M81141 [36773\_f\_at(59, 225) ] | 5 | AD334T2 \_A280\_10(CL2001032625AA) (0.71) [9, 12] | ADA31\_A289\_10(CL2001032259AA) (3.19) [7, 5] | AD208T1\_A143\_4(CL2001032229AA) (1.0)[6, 1] |
| U41635 [36996\_at(5, 433) ] | 14 | AD268T2\_A189\_4(CL2001032633AA) (0.95) [9, 14] | AD375T1\_A115\_3(CL2001032322AA) (2.16) [8, 7] | AD258T1\_A170\_4(CL2001032646AA) (1.0)[10, 8] |
| X59417 [36122\_at(208, 285) ] | 1 | AD338T1\_A121\_3(CL2001032115AA) (0.59) [4, 7] | ADA2T1\_A384\_7(CL2001032842AA) (3.49) [13, 2] | AD347T1\_A134\_3(CL2001032243AA) (1.0)[6, 8] |
| X69433 [32332\_at(186, 81) ] | 10 | AD226T2\_A151\_4(CL2001031618AA) (0.8) [1, 7] | AD374T1\_A114\_3(CL2001032248AA) (3.0) [6, 12] | AD383T1\_A118\_3(CL2001031631AA) (1.0)[1, 14] |
| U14968 [32436\_at(2, 177) ] | 15 | AD136T2\_A201\_8(CL2001032729AA) (0.21) [11, 8] | AD120T2\_A196\_8(CL2001032730AA) (5.68) [11, 9] | AD259T1\_A171\_4(CL2001032213AA) (1.23)[5, 11] |
| U79275 [36266\_at(279, 159) ] | 14 | AD157T2\_A26\_1(CL2001032242AA) (0.9) [6, 7] | AD119T3\_A195\_8(CL2001032845AA) (2.53) [13, 5] | Null |
| Y08976 [31685\_at(527, 149) ] | 11 | AD269T1\_A94\_3(CL2001031635AA) (0.64) [2, 3] | AD360T1\_A176\_4(CL2001032639AA) (2.74) [10, 4] | AD334T1 \_A221\_8(CL2001032707AA) (1.0)[11, 1] |
| Z49148 [33674\_at(535, 299) ] | 12 | AD336T1\_A223\_8(CL2001041708AA) (0.96) [14, 2] | AD353T1\_A129\_3(CL2001032857AA) (2.6) [13, 9] | Null |
