# Supplementary material for: AffyMAPSDetector: a software tool to characterize Affymetrix GeneChip™ expression arrays with respect to SNPs
Source: BMC Bioinformatics. 2007 Jul 30;8:276. doi: 10.1186/1471-2105-8-276 (PMC1959249; doi:10.1186/1471-2105-8-276)

### Additional file 10 – Example probes affecting probe-set detection calls.

File format – Microsoft Word (use MS word for Microsoft Windows and Open Office for Microsoft Windows, GNU/Linux ("Linux"), Sun Solaris, Mac OS X (under X11), and FreeBSD; Open office is an open source software, downloadable from http://www.openoffice.org/).

Description – This file contains some examples of SNP-containing probes that affect probe-set detection call.

**Probe-set ID:** 41409_at

**Sample:** CL2001031611AA

Comparison of the original and the modified probe intensity profiles (when roles of PM and MM probes are swapped at position 6 and 7) indicate improvement in transcript abundance signal computation. Note that on the left panels, blue color shows the PM probes’ profile and green color shows the MM probes’ profile.


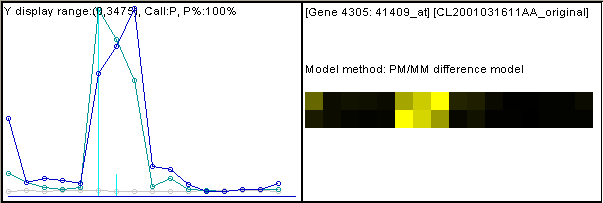

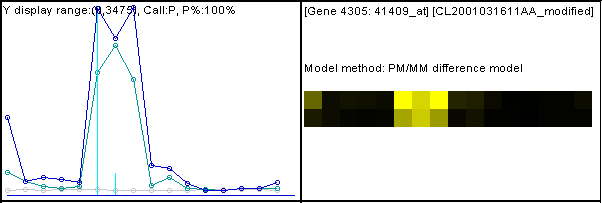


**Probe-set ID:** 1486_at

**Sample:** CL2001031611AA

Comparison of the original and the modified probe intensity profiles (when roles of PM and MM probes are swapped at position 12) indicate improvement in transcript abundance signal computation as well as the P-call changes from Absent to present.


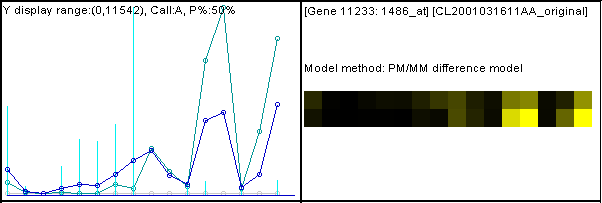


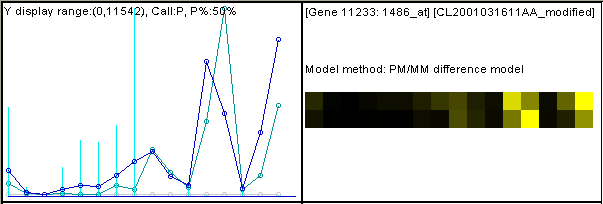


**Probe-set ID:** 32001_s_at

**Sample:** CL2001031611AA

Comparison of the original and the modified probe intensity profiles (when roles of PM and MM probes are swapped at position 10) indicate improvement in transcript abundance signal computation.


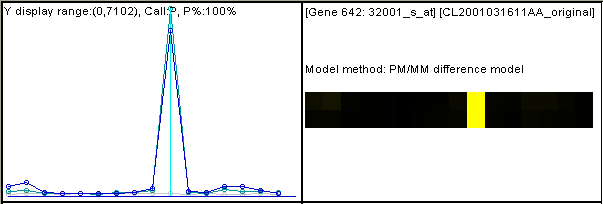

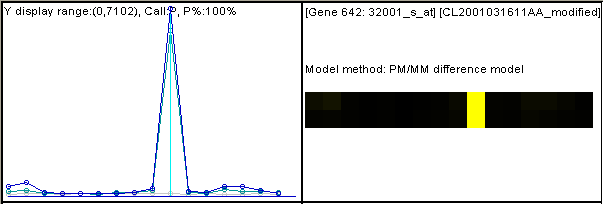


**Probe-set ID:** 32313_at

**Sample:** CL2001031611AA

Comparison of the original and the modified probe intensity profiles (when roles of PM and MM probes are swapped at position 8) indicate improvement in transcript abundance signal computation.


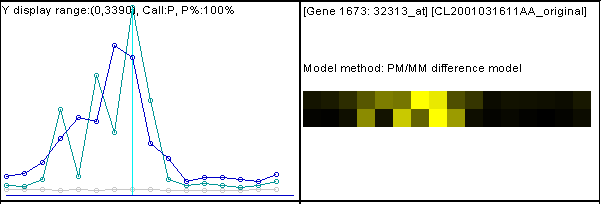

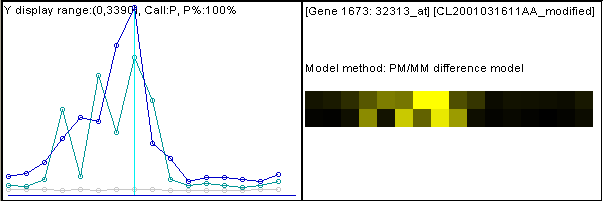


**Probe-set ID:** 34345_at

**Sample:** CL2001031611AA

Comparison of the original and the modified probe intensity profiles (when roles of PM and MM probes are swapped at position 14 and 15) indicate improvement in transcript abundance signal computation as well as P-call changes from Absent to Present.


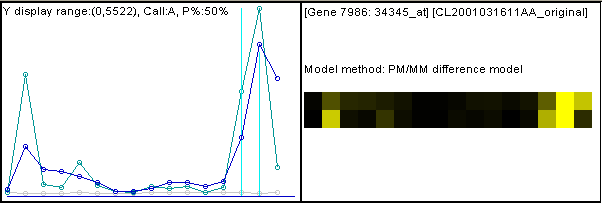

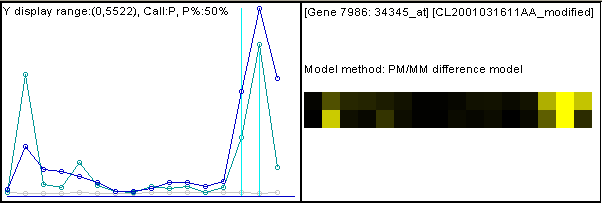


**Probe-set ID:** 36644_at

**Sample:** CL2001031611AA

Comparison of the original and the modified probe intensity profiles (when roles of PM and MM probes are swapped at position 16) indicate improvement in transcript abundance signal computation.


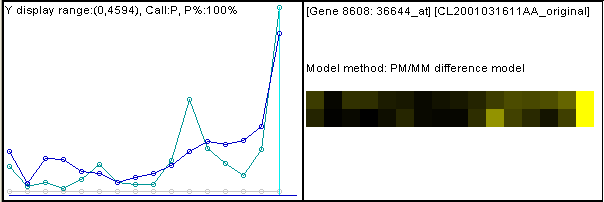

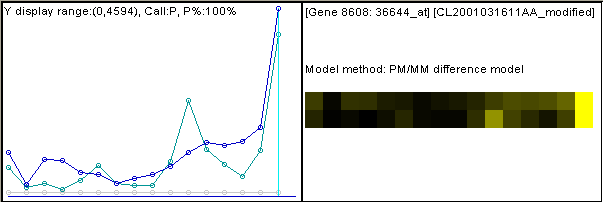


**Probe-set ID:** 36661_s_at

**Sample:** CL2001031611AA

Comparison of the original and the modified probe intensity profiles (when roles of PM and MM probes are swapped at position 13 and 15) indicate improvement in transcript abundance signal computation.


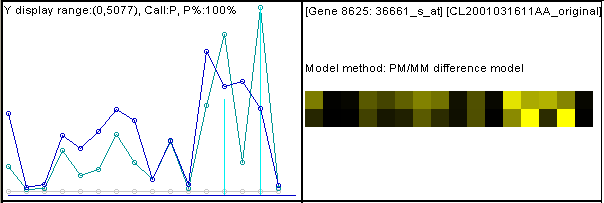


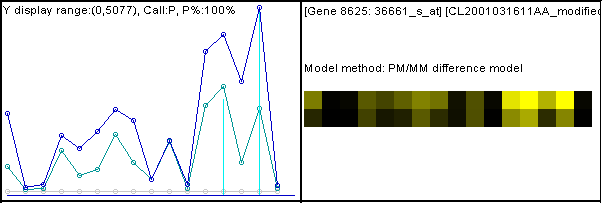


**Probe-set ID:** 36878_f_at

**Sample:** CL2001031611AA

Comparison of the original and the modified probe intensity profiles (when roles of PM and MM probes are swapped at position 5) indicate that in such cases (when both intensity values are close enough) there would hardly be any improvement in the transcript abundance signal computation.


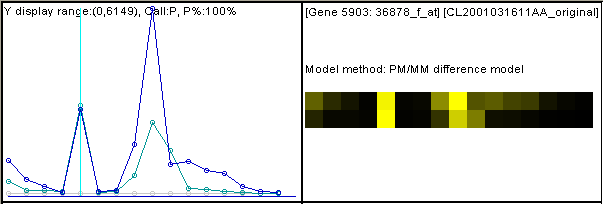

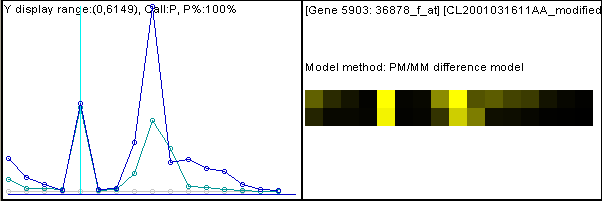


**Probe-set ID:** 37746_r_at

**Sample:** CL2001031611AA

Comparison of the original and the modified probe intensity profiles (when roles of PM and MM probes are swapped at position 9 and 15) indicate improvement in transcript abundance signal computation as well as the P-call changes from Marginal to Present.


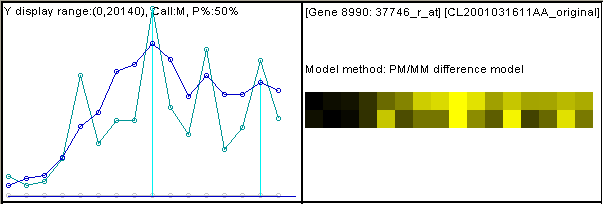

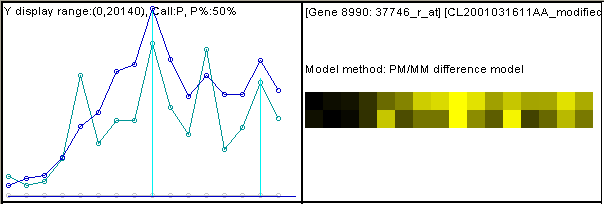


**Probe-set ID:** 38391_at

**Sample:** CL2001031611AA

Comparison of the original and the modified probe intensity profiles (when roles of PM and MM probes are swapped at position 14) indicate improvement in transcript abundance signal computation.


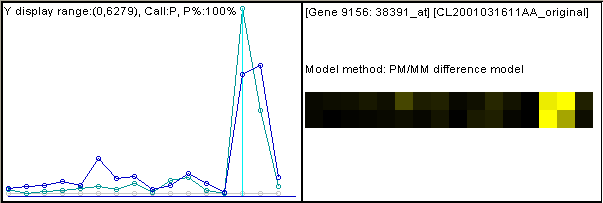


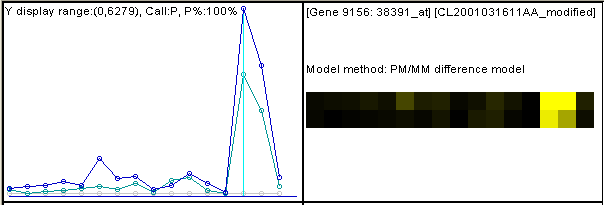


**Probe-set ID:** 39069_at

**Sample:** CL2001031611AA

Comparison of the original and the modified probe intensity profiles (when roles of PM and MM probes are swapped at position 13) indicate improvement in transcript abundance signal computation.


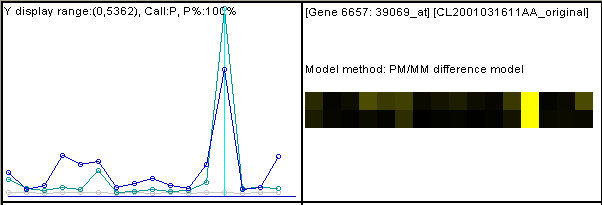

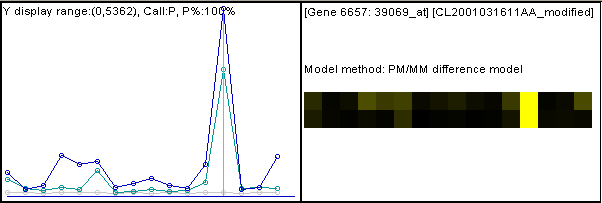


**Probe-set ID:** 40794_at

**Sample:** CL2001031611AA

Comparison of the original and the modified probe intensity profiles (when roles of PM and MM probes are swapped at position 10 and 11) indicate improvement in transcript abundance signal computation.


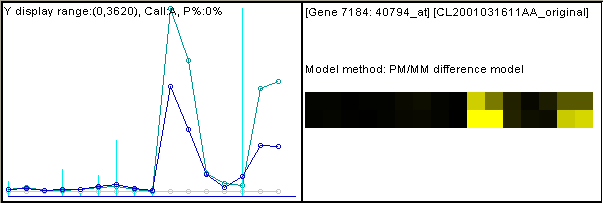

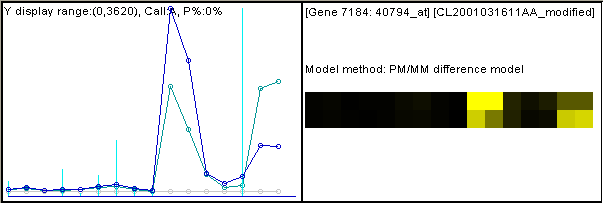


**Probe-set ID:** 41773_at

**Sample:** CL2001031611AA

Comparison of the original and the modified probe intensity profiles (when roles of PM and MM probes are swapped at position 7 and 10) indicate improvement in transcript abundance signal computation.


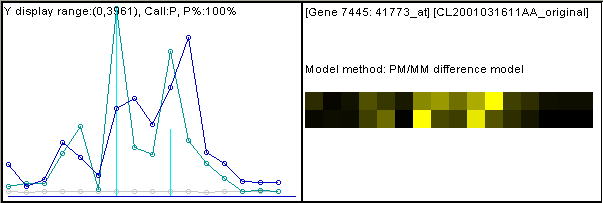

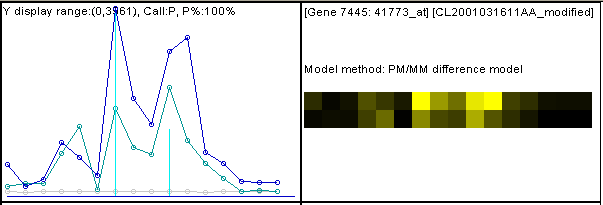

Supplement: Additional file 10 — Example of probes affecting probeset detection calls. This file contains some examples of SNP-containing probes that affect probeset detection call. [file 1471-2105-8-276-S10.doc]
