# Supplementary figures and images for: AffyMAPSDetector: a software tool to characterize Affymetrix GeneChip™ expression arrays with respect to SNPs
Source: BMC Bioinformatics. 2007 Jul 30;8:276. doi: 10.1186/1471-2105-8-276 (PMC1959249; doi:10.1186/1471-2105-8-276)

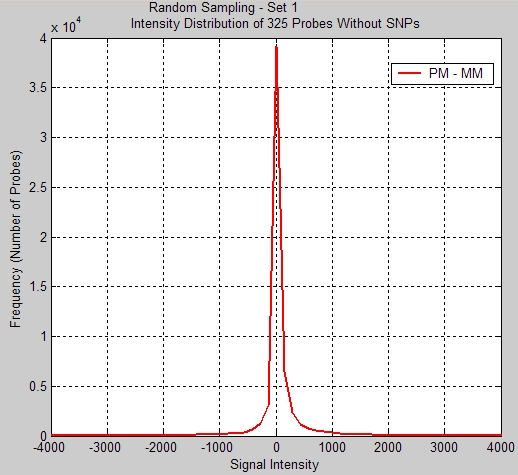

Supplement: Additional file 14 — Intensity distribution profiles' confirmation. The set of files in this archive contain data analysis experiment results on lung adenocarcinoma dataset to confirm repeatability of intensity distribution patterns differences between probes with SNPs and probes without SNPs. [file 1471-2105-8-276-S14.zip › 325_samples_no_snps_intensity_dist_1.jpg]

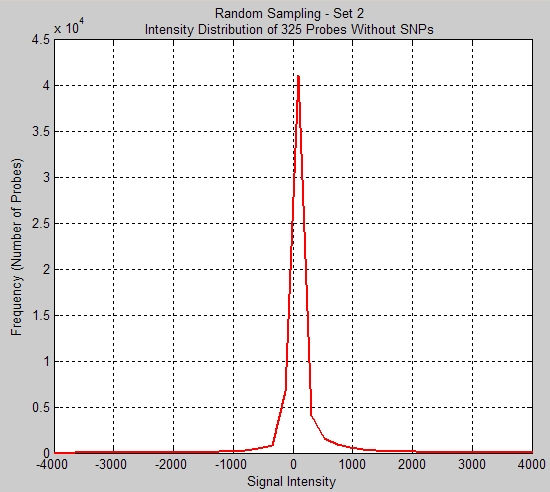

Supplement: Additional file 14 — Intensity distribution profiles' confirmation. The set of files in this archive contain data analysis experiment results on lung adenocarcinoma dataset to confirm repeatability of intensity distribution patterns differences between probes with SNPs and probes without SNPs. [file 1471-2105-8-276-S14.zip › 325_samples_no_snps_intensity_dist_2.jpg]

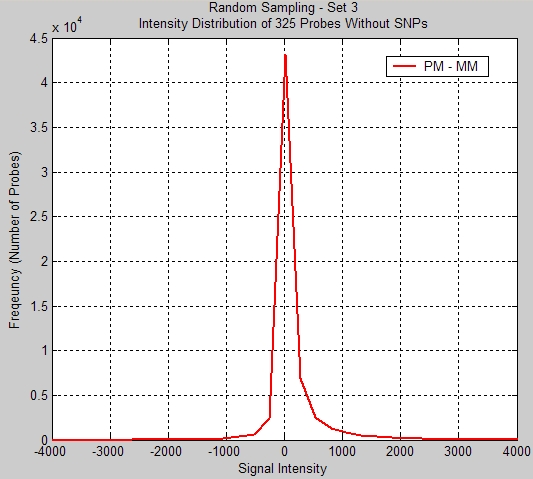

Supplement: Additional file 14 — Intensity distribution profiles' confirmation. The set of files in this archive contain data analysis experiment results on lung adenocarcinoma dataset to confirm repeatability of intensity distribution patterns differences between probes with SNPs and probes without SNPs. [file 1471-2105-8-276-S14.zip › 325_samples_no_snps_intensity_dist_3.jpg]

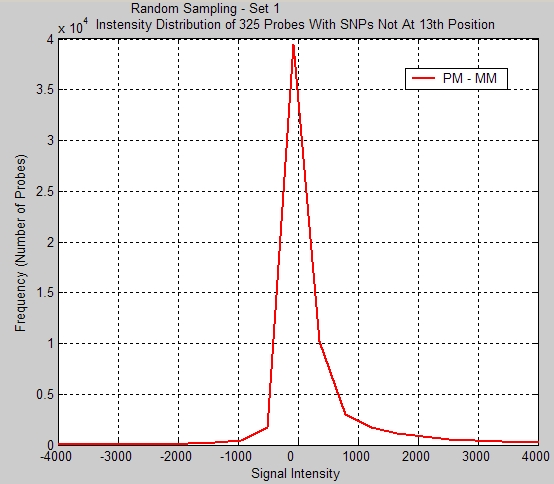

Supplement: Additional file 14 — Intensity distribution profiles' confirmation. The set of files in this archive contain data analysis experiment results on lung adenocarcinoma dataset to confirm repeatability of intensity distribution patterns differences between probes with SNPs and probes without SNPs. [file 1471-2105-8-276-S14.zip › 325_samples_snps_not_at_13_intensity_dist_1.jpg]

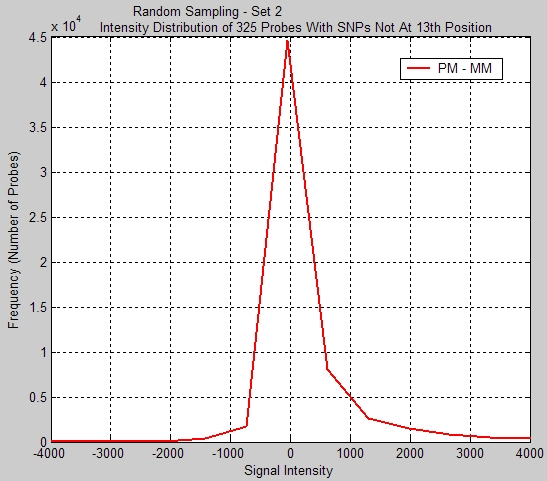

Supplement: Additional file 14 — Intensity distribution profiles' confirmation. The set of files in this archive contain data analysis experiment results on lung adenocarcinoma dataset to confirm repeatability of intensity distribution patterns differences between probes with SNPs and probes without SNPs. [file 1471-2105-8-276-S14.zip › 325_samples_snps_not_at_13_intensity_dist_2.jpg]

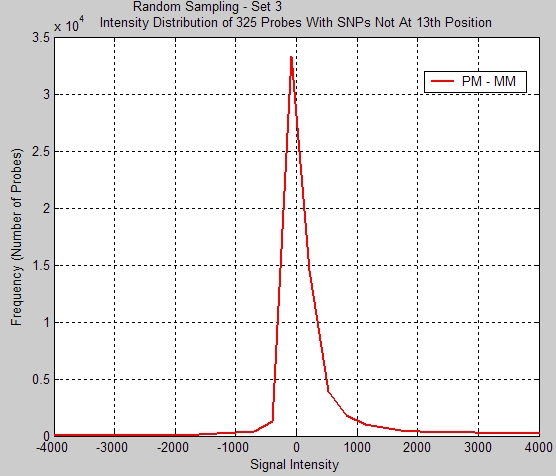

Supplement: Additional file 14 — Intensity distribution profiles' confirmation. The set of files in this archive contain data analysis experiment results on lung adenocarcinoma dataset to confirm repeatability of intensity distribution patterns differences between probes with SNPs and probes without SNPs. [file 1471-2105-8-276-S14.zip › 325_samples_snps_not_at_13_intensity_dist_3.jpg]
